# An economic evaluation of eptinezumab for the preventive treatment of migraine in the UK, with consideration for natural history and work productivity

**DOI:** 10.1186/s10194-024-01749-8

**Published:** 2024-04-18

**Authors:** Edward Griffin, Gawain Shirley, Xin Ying Lee, Susanne F. Awad, Alok Tyagi, Peter J. Goadsby

**Affiliations:** 1 Exeter, UK; 2 Lundbeck Ltd, Watford, UK; 3 H. Lundbeck A/S, Copenhagen, Denmark; 4 NHS Greater Glasgow and Clyde, Scotland, UK; 5 SLaM Biomedical Research Centre, NIHR King’s Clinical Research Facility, and Wolfson SPaRC, King’s College London, London, UK

**Keywords:** Eptinezumab, Anti-CGRP mAbs, Migraine, Cost-effectiveness, Healthcare costs, Natural history, Productivity, UK

## Abstract

**Background:**

Migraine is a highly prevalent neurological disease with a substantial societal burden due to lost productivity. From a societal perspective, we assessed the cost-effectiveness of eptinezumab for the preventive treatment of migraine.

**Methods:**

An individual patient simulation of discrete competing events was developed to evaluate eptinezumab cost-effectiveness compared to best supportive care for adults in the United Kingdom with ≥ 4 migraine days per month and prior failure of ≥ 3 preventive migraine treatments. Individuals with sampled baseline characteristics were created to represent this population, which comprised dedicated episodic and chronic migraine subpopulations. Clinical efficacy, utility, and work productivity inputs were based on results from the DELIVER randomised controlled trial (NCT04418765). Timing of natural history events and treatment holidays—informed by the literature—were simulated to unmask any natural improvement of the disease unrelated to treatment. The primary outcomes were monthly migraine days, migraine-associated costs, quality-adjusted life years (QALYs), incremental cost-effectiveness ratio, and net monetary benefit, each evaluated over a 5-year time horizon from 2020. Secondary analyses explored a lifetime horizon and an alternative treatment stopping rule.

**Results:**

Treatment with eptinezumab resulted in an average of 0.231 QALYs gained at a saving of £4,894 over 5 years, making eptinezumab dominant over best supportive care (i.e., better health outcomes and less costly). This result was confirmed by the probabilistic analysis and all alternative assumption scenarios under the same societal perspective. Univariate testing of inputs showed net monetary benefit was most sensitive to the number of days of productivity loss, and monthly salary.

**Conclusions:**

This economic evaluation shows that from a societal perspective, eptinezumab is a cost-effective treatment in patients with ≥ 4 migraine days per month and for whom ≥ 3 other preventive migraine treatments have failed.

**Trial registration:**

N/A.

**Graphical Abstract:**

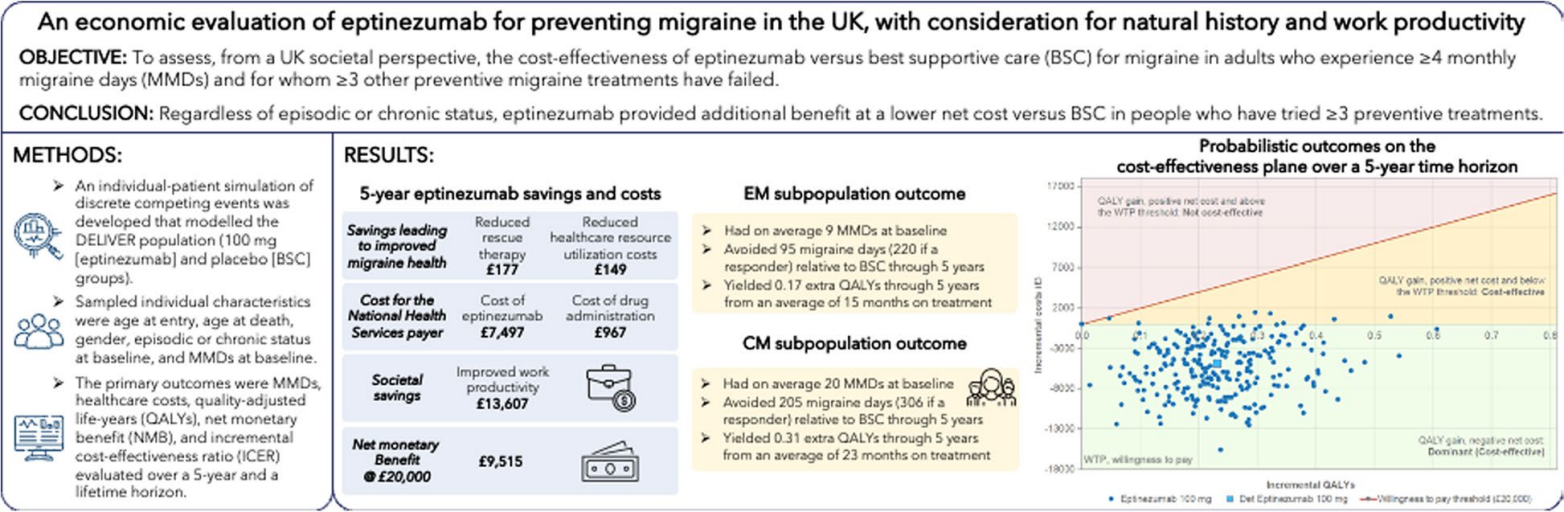

**Supplementary Information:**

The online version contains supplementary material available at 10.1186/s10194-024-01749-8.

## Background

Migraine is a highly prevalent neurological disease of many types but is primarily characterised by recurrent episodes of primary headache [1]. It was ranked in the top ten causes of disability among people aged 10–49 years in the Global Burden of Disease study (1990–2019) [2]. In England in 2019, age-standardised prevalence and incidence rates were reported as 14.7% for total prevalent cases and 0.3% for total new cases, respectively [3]. Migraine is more common in women than men, with 75% of migraineurs being female in a recent study on the sex differences in migraine prevalence [4]. The prevalence peak is observed during an age of prime productivity (30–40 years) and decreases with age regardless of gender [5].

Apart from the direct burden to the patients, migraine has a substantial burden and cost to society. In addition to classical migraine symptoms, migraine is associated with an increased risk of experiencing sleep-related problems, depression, and anxiety [6–11]. This may substantially affect patients’ ability to work and/or learn effectively, with subsequent detrimental effects on their own career and financial stability, diminishing their potential to contribute to the economy as a whole [12, 13]. A study conducted by the Work Foundation estimated that 86 million workdays are lost to migraine-related absenteeism and presenteeism in the United Kingdom (UK) each year, at a cost to society of £8.8 billion [14]. In a real-world study including patients with migraine in Europe, patients treated with 3 or more preventive migraine treatments had significantly worse Work Productivity and Activity Impairment (WPAI) scores than patients who had never received a preventive migraine treatment [15]. WPAI scores indicated more frequent migraine is associated with higher rates of absenteeism and presenteeism, and greater work productivity and activity impairment.

According to the International Classification of Headache Disorders (third edition), chronic migraine (CM) is defined as headache occurring on 15 or more days per month for more than three months, where at least eight of those headache days have migraine features [1]. Episodic migraine (EM) has been described in an amendment to the glossary as having less than 15 monthly headache days (MHDs) where some are migraine [16].

Eptinezumab is an anti-calcitonin gene-related peptide monoclonal antibody (anti-CGRP mAb) administered intravenously (IV) every 12 weeks. It joins three subcutaneously administered options within the same class that were already available to patients. DELIVER was the pivotal trial supporting the 2023 recommendation for reimbursement of eptinezumab (100 mg) by the National Institute for Health and Care Excellence (NICE) and Scottish Medicines Consortium. The trial was a 24-week multi-centre, double-blind, multi-arm, placebo-controlled, randomised phase 3b trial designed to investigate the safety and efficacy of eptinezumab for migraine prevention in patients with two to four prior preventive treatment failures, in which patients were assigned 1:1:1 to eptinezumab 100mg, eptinezumab 300mg, or placebo (0.9% saline) [17]. The DELIVER trial showed that improvements in absenteeism and presenteeism were greater at all timepoints in the eptinezumab groups when compared to placebo [18].

The anti-CGRP mAbs are all indicated for the preventive treatment of migraine in adults who have at least four monthly migraine days (MMDs) [19], which was the minimum threshold for the definition of EM in DELIVER and the NICE technology appraisals of anti-CGRP mAbs [20–23]. Additionally, in the UK, it is recommended that people have tried and failed three other preventive treatments before being eligible for anti-CGRP mAb treatment [23]. Whilst NICE determined eptinezumab to be cost effective from the National Health Service (NHS) and the personal and social services perspective in England [24], cost-effectiveness using a wider perspective that includes economic productivity was not evaluated. The creation of Integrated Care Systems and the development of partnerships between health services and local authorities to tackle the wider determinants of health, such as employment and education, means that economic productivity is increasingly an outcome of interest [25, 26].

The aim of the current study is to inform joined-up healthcare decision-making by assessing, from a societal perspective, the cost-effectiveness of eptinezumab versus best supportive care (BSC), for adults that have four or more MMDs and who have tried three or more other preventive treatments that failed. Despite a growing number of treatment options for this population, BSC remains a relevant comparator [27].

## Methods

### Population

The modelled population, referred herein as the TF3+ population, was adults who received either eptinezumab 100mg or placebo during the DELIVER study, have at least four MMDs, and who have tried three or more other preventive treatments that failed. The 300-mg arm was excluded since 300-mg vials are unavailable in the UK. This TF3+ population represents 25.6% (228/890) of the trial final analysis set, of which 51% received eptinezumab 100mg and 49% placebo. It comprised two subpopulations, individuals classified with either EM or CM. Reflecting the International Classification of Headache Disorders (3rd edition [1]) and DELIVER definitions, CM was defined in this analysis as at least 15 MHDs where at least eight have migraine features. EM was defined as up to 14 MHDs where at least four have migraine features. Since a headache can also be a migraine, these definitions do not preclude the overlapping of MMD distributions across respective subpopulations, as was seen in TF3+ subgroup of DELIVER. However, from the commencement of treatment at model entry, change in migraine burden was a function only of MMD frequency, not MHD frequency, which was not tracked. This was particularly relevant to improvement from CM to EM in the modelling of natural history. In any case, baseline values in the TF3+ subgroup of DELIVER showed a ratio of 21 MHDs to 20 MMDs, indicating that the number of MHDs was largely driven by the number of MMDs. This approach then relaxes the strict definition of subpopulations. This is common to migraine models but is additionally relevant in this simulation because subpopulation status was allowed to change with natural improvement in MMDs, necessitating an MMD-based boundary. Nonetheless, the modelled outcomes for subpopulations use the EM/CM label as defined and attributed at baseline.

At model entry, the sampled individual characteristics were age at entry (mean 45.4, range 18 to 85), life expectancy, gender, subpopulation status, and MMDs. Age at entry, gender, and subpopulation MMDs at baseline were sampled based on a post hoc analysis of the TF3+ subgroup of DELIVER. Age at death was calculated from life expectancy, which was sampled using national life tables for England [28]. Subpopulation at baseline was sampled using a flat distribution of 46% with chronic migraine. To improve external validity in this defining input, the estimate was based on a UK market research survey of MMD frequency in people with a diagnosis of migraine in the UK population (data on file). This compares to 49% in the TF3 + subgroup of DELIVER. More detail of the sampling distributions is provided in the Supplemental Methods.

### Model structure

An individual patient simulation model was developed in Microsoft Excel 365 to create and simulate migraine-related events over the lifetime of 5,000 unique individuals with migraine and eligible for treatment with eptinezumab 100 mg. In testing, five-thousand microsimulations ensured stochastic stability in mean per person costs and QALYs. In the micro-simulation of every unique life, the accumulation of quality-adjusted life-years (QALYs) and the consumption of healthcare resources were tracked and averaged across the whole population to inform eptinezumab and BSC strategy outcomes. The health outcomes for individuals, when run through the BSC strategy, were based on the placebo arm of the DELIVER trial, and so represents the collective outcome of loosely described sequential and concomitant use of acute medications. Cost-effectiveness was examined using the incremental cost-effectiveness ratio (ICER) and net monetary benefit at a willingness-to-pay threshold of £20,000 per QALY gained. This is the lower of the two standard thresholds considered by NICE (£20,000 and £30,000 per QALY gained) [24]. The analysis took a societal perspective for the NHS using a 2020 cost year, therefore including wider economic costs associated with impaired work productivity.

The model used a discrete event simulation framework, an approach which is conceptualised around the occurrence and timing of events. It is well suited for implementing more complex models which demand a high number of health states and/or deal with input parameters as continuous variables but represent disease progression as a series of discrete events [29]. In migraine, the MMD range 0–30 represents a high number of health states; baseline severity and treatment improvement measured in MMDs represents the continuous variable; and natural transformation and resolution represent discrete events on the MMD continuum. Beyond this, the framework also offers the flexibility for capturing the large number of possible migraine events that cover natural history changes, multiple causes of discontinuation, and a schedule of treatment holidays. The cohort-based transition models that have been adopted for previous technology appraisals assess changes over fixed time intervals, which can present a difficulty when some events repeat multiple times or occur with both small and large intervals. In this simulation, a model clock is moved forward in time to the point at which the next event and new clinical state is experienced. Clinical states are an additional secondary structure allowing the time horizon to be measured in terms of definable periods of clinical management (e.g., assessing response or treatment holiday periods). This modelling approach is new to migraine but facilitates the inclusion of natural history and the clinical framework of treatment holidays to identify any change this may have on the burden and progression of migraine. Notwithstanding the above, the assumptions and input preferences of prior NICE technology appraisals of anti-CGRP mAbs were taken forward.

**Fig. 1:**
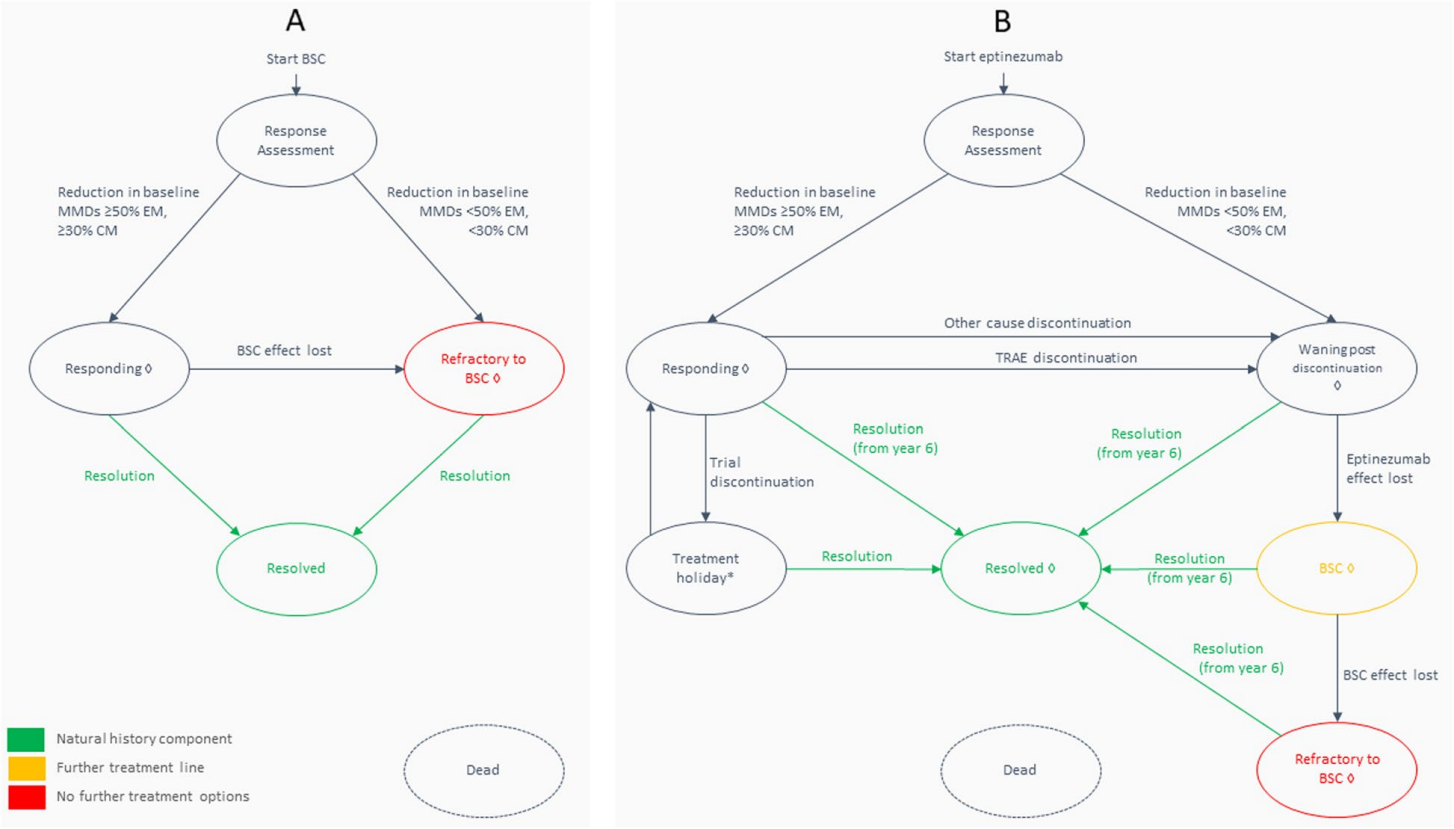
Model structure depicted as clinical states and permitted paths with (**A**) best supportive care (BSC) and (**B**) eptinezumab. Key: Ovals are clinical states. Arrows represent a permitted movement to or from a clinical state. Dashed ovals are clinical states with entry allowed from any other clinical state. * 3-month treatment holiday for assessment of natural improvement (maximum of five, 12 months between). ◊ Clinical state where natural improvement is permitted after five treatment holiday cycles (see methods for details of how natural history was applied)

Figure 1 depicts the clinical paths and states for the competing strategies of BSC and eptinezumab from model entry (i.e., the point at which the simulation starts running for the individual patient). The permitted paths common to the considered strategies were negative discontinuation and natural improvement in the underlying condition. Negative discontinuation was applied when MMD change over 12 weeks failed to meet the response rate criteria of ≥ 50% reduction in MMDs in EM or ≥ 30% reduction in MMDs in CM, relative to before starting treatment. Underlying improvement could occur through a transformation event, where MMD frequency was reduced from chronic to episodic severity. Also, by result of a resolution event, where MMD frequency was reduced to below the defined EM range and the MMD threshold for starting preventive treatment [30]. Otherwise, strategies differed in their permitted clinical paths. The eptinezumab strategy considered treatment-emergent adverse events (TEAEs) leading to eptinezumab discontinuation, discontinuation from other causes, and a schedule of holidays from active treatment. A further line of treatment, BSC, followed any discontinuation event in the eptinezumab strategy. As a subsequent line, the BSC treatment effect was independent of response to prior eptinezumab, resembling the modelled approach of BSC as a comparator. A scenario examined the impact of eliminating BSC as a subsequent treatment line.

### Treatment effect

The DELIVER trial is currently the only study that investigated the efficacy and safety of eptinezumab versus BSC (placebo) in patients with prior failure of preventive migraine treatment [17]. However, efficacy data for the modelled population exclude participants of DELIVER receiving eptinezumab 300mg, and those failing less than three prior preventive treatments to reflect the reimbursed dose and population for eptinezumab in the model setting. Response rates for the remaining cohort were imputed into the model, and these were defined as ≥ 50% MMD reduction for individuals with EM and ≥ 30% MMD reduction for individuals with CM. The respective mean change in MMDs over weeks 1–12 was imputed at subpopulation and response status level. These inputs are presented in Table 1, including an alternative response threshold of ≥ 50% MMD reduction in the CM subpopulation.

**Table 1:** Efficacy inputs for migraine treatments in the economic model

Results over 12 weeks of treatment, with standard error (SE)	TF3+ EM, ≥ 50% reduction in MMDs	TF3+ CM, ≥ 30% reduction in MMDs	TF3+ CM, ≥ 50% reduction in MMDs (scenario)
Responder rate, BSC, %	9.6 (2.0)	23.2 (4.6)	8.9 (1.8^a^)
Responder rate, eptinezumab 100mg, %	40.0 (8.0)	63.4 (12.9)	30.4 (6.1^a^)
Change from baseline in MMDs, BSC responders	-5.82 (1.50)	-8.80 (1.99)	-10.45 (1.41)
Change from baseline in MMDs, eptinezumab 100 mg responders	-6.82 (0.92)	-9.93 (1.41)	-12.68 (1.40)
Change from baseline in MMDs, BSC non-responders	-1.31 (0.84)	-0.85 (1.42)	-1.94 (0.96)
Change from baseline in MMDs, eptinezumab 100 mg non-responders	-1.39 (1.18)	-1.71 (2.28)	-4.52 (1.17)

*Key* BSC, best supportive care, *CM* chronic migraine, *EM* episodic migraine, *MMDs* monthly migraine days, *SE* standard error, *TF3*+ have tried three or more other preventive treatments that failed

^a^SE = 20% of mean estimate

The mean change in MMDs over weeks 1–12 was applied to the baseline frequency according to treatment strategy (eptinezumab/BSC), subpopulation status at baseline (EM/CM), and response status (responder/non-responder). In the absence of events leading to discontinuation, mean improvement at week 12 was maintained. No advantage in speed of onset was conveyed to eptinezumab over BSC, although response has been observed as early as the day following the first eptinezumab infusion [17]. Similarly, the rate of improvement through the first 12 weeks was assumed linear in all cases for both treatments.

Positive response over 12 weeks was defined as a reduction in MMDs of at least 50% in EM and at least 30% in CM according to NICE recommendations in the UK [20–22]. A secondary analysis explored the more stringent definition of positive response for CM as a reduction in MMDs of at least 50%. Responders to eptinezumab maintained the improvement reached over 12 weeks until death or treatment discontinuation. All discontinuation events triggered a four-month linear wane of effect to baseline MMDs [31]. Based on clinician input toward the open-question of disease-modification from long-term treatment in individuals with a long history of migraine [32], it was assumed that 10% of responding patients who enter a treatment holiday maintained the on-treatment improvement and did not restart treatment (a ‘super-response’ leading to ‘positive stopping’). Responders to BSC effectively discontinued treatment after one year, when the treatment effect experienced by 12 weeks was diminished to baseline after a process of linear waning. This assumption was based on NICE preferences in prior CGRP mAb appraisals [20, 22].

Non-responders to eptinezumab did not receive further eptinezumab infusions and were assumed to subsequently receive BSC. In the BSC strategy, the treatment effect attributed to both responders and non-responders waned linearly to baseline frequency over one year [20]. Age- and gender-adjusted mortality was included in the model, but this was not affected by choice of treatment strategy.

### Natural history

Change in the natural history of migraine through its course has been documented and measured in longitudinal studies [33, 34]. Fluctuations in EM and CM status are reported even over a single year [35]. However, only sustained improvement was considered since the modelled population is already defined as having considerable migraine and treatment history. In this predominantly female population, the basis of natural improvement was menopause. This approximated the timing of the diminishing prevalence of migraine for both men and women and was applied in the model through transformation and resolution events [36, 37]. Transformation described a remission from CM to EM status and was equal to the sampled age of menopause, so the timing varied between individuals Note that for individuals who entered the model already older than their sampled age at menopause, natural improvement events were not possible. Resolution described a remission from EM to sub-therapeutic frequency migraine (i.e., three or less MMDs). For individuals entering the model with chronic migraine, resolution first required transformation. Since change in migraine health in the model is a function only of MMD frequency not MHD frequency, the CM subpopulation MMD boundary required special definition. With the benefit of expert clinical advice, this was set at eight MMDs, and transformation reduced MMDs to seven in every effected individual. Similarly, resolution reduced MMDs to three in all cases, a sub-therapeutic frequency. Time at transformation was sampled using a normal distribution around mean age of onset of menopause (mean [SD], 49.5 [5.0] years [38]); and resolution was 4.3 years later, based on the mean length of menopause symptoms [39]. Natural improvement events were detected at the next scheduled treatment holiday. The schedule of consisted of five 15-month cycles comprising 12 months on-treatment [32] followed by a 3-months off-treatment holiday, totalling 75 months, after which it was assumed that natural history events were no longer confined to treatment holidays. In the model, no patients remained on treatment by 5 years. Detail of sampling distributions are provided in the Supplemental Methods.

### Treatment discontinuation

**Fig. 2:**
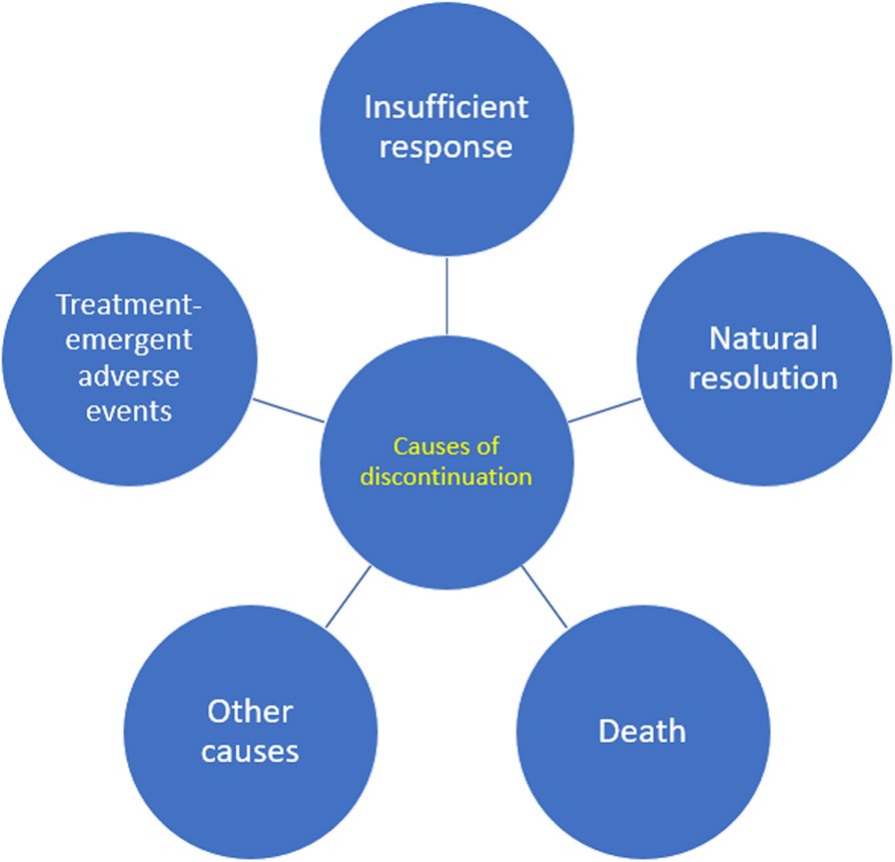
Modelled modes of discontinuation of eptinezumab

Both eptinezumab and BSC treatment were discontinued consequent to death or the negative stopping rule, which was applied to patients with insufficient response over 12 weeks. Eptinezumab could be discontinued at any time due to TEAEs. The risk of a TEAE leading to discontinuation during the assessment period was based on rates in DELIVER, and the 2-year open-label PREVAIL study thereafter [40]. Discontinuation also followed natural resolution when migraine frequency fell below the therapeutic range. This mode of discontinuation was effectively limited to the eptinezumab strategy since a year of preventive treatment was required before the first treatment holiday. Finally, other-cause discontinuation (e.g., due to patient preference) was included as a catch-all mode of discontinuation used in the model to calibrate the overall rate of discontinuation against real-world evidence of subcutaneous anti-CGRP mAb discontinuation rates in Sweden (data on file). Figure 2 illustrates the competing modes of eptinezumab discontinuation within the model. Detail of sampling of discontinuation times are provided in the Supplemental Methods.

### Health-related quality of life

At any time in a simulated patient’s life, including periods of being on- and off-treatment, health-related quality of life was applied as a utility score of 0–1, where zero represents death and one represents full health. The utility score was a function of MMDs and whether treatment was active with eptinezumab at the time.

The Migraine-Specific Quality of life questionnaire (MSQ, 2.1) was used in DELIVER to measure health-related quality of life at baseline, week 12 and week 24 [41]. MSQ scores from the TF3+ cohort were mapped to EQ-5D-3L scores and converted to utilities using UK population-based tariffs and the mapping algorithm described by Gillard et al. [42, 43]. Linear regression of utility score and MMDs identified a statistically significant relationship, and a difference in utility between individuals on- and off- eptinezumab. Linear type regression has been consistently adopted by NICE in GCRP mAb appraisals [20–22], and differential utility was also preferred in the most recent of these [20]. This utility regression analysis for the DELIVER intended to treat population has been published separately [44]. For the TF3+ population of DELIVER, utility in patients with one MMD was 0.762 when on treatment with eptinezumab and 0.707 when not. An additional disutility of 0.013 was applied for each additional MMD. This was carried forward into the model, standardising for age and gender [45]. No disutility for intravenous infusion of eptinezumab was included since this was considered uncertain, infrequent, and too short-lived to impact QALYs over the time horizon.

### Resources and costs

Consumption of health system resources other than drug acquisition and administration depended on MMD frequency. Eptinezumab was administered at 12-week intervals; the unit cost of 100 mg vials was £1,350, which is the published listed price of eptinezumab [46]. Infusions were in the hospital setting and cost £171.04 per infusion. This infusion cost was based on IV administration costs of infliximab for rheumatoid arthritis, inflated to the 2020 cost-year [47]. For simplicity, the acquisition and administration costs of BSC were excluded, given that they are generally low in NHS acquisition cost and are predominantly administered orally.

Disease management resources were categorised to general practitioner and nurse practitioner consultations in primary care, emergency and elective hospital episodes, neurologist and psychiatrist consultations, and instances of triptan medical rescue. Rates of consumption were sourced from the 2021 update of the 2016 National Health and Wellbeing Survey within France, Germany, Italy, Spain, and the UK (data on file). The National Schedule of NHS costs 2019/20 and the PSSRU unit costs of Health & Social Care 2020 were used for unit costing (both prior to the Covid-19 pandemic) [48, 49]. Further detail of unit costs and consumption rates are provided in the Supplemental Methods.

### Work productivity

**Fig. 3:**
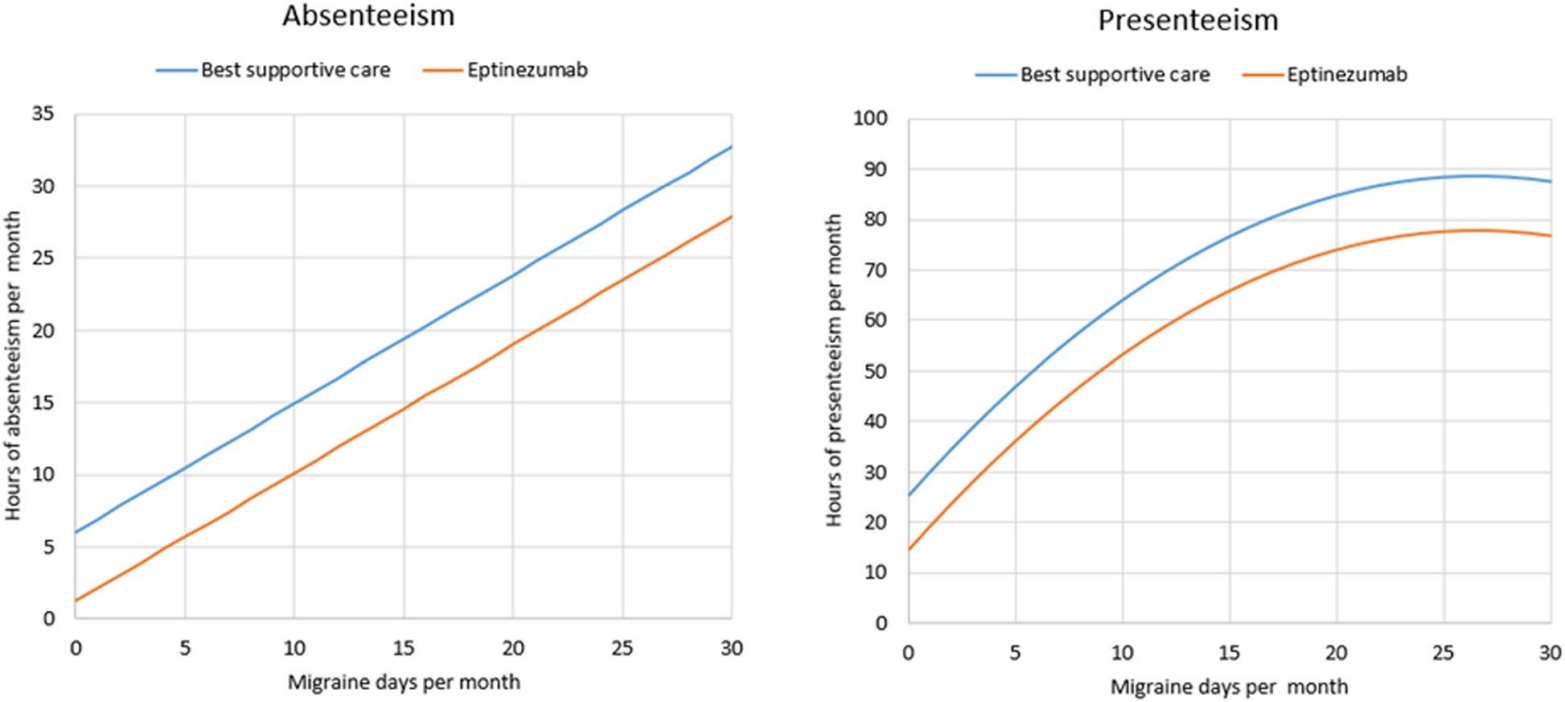
Self-reported migraine-related impairment of work productivity in the full DELIVER population (2–4 prior treatment failures) over 24 weeks, by administered treatment

The economic cost of lost productivity from human capital was estimated by regressing the number of MMDs against patient-reported absenteeism and presenteeism scores from the WPAI questionnaire. Data from the full DELIVER cohort over 24 weeks was taken forward to maximise the number of observations in a regression analysis of hours affected by absenteeism and presenteeism versus migraine day frequency. The relationship over the first 24 weeks were assumed generalisable to the full model time horizon [50]. Figure 3 shows the linear and quadratic regression functions, explored by administered treatment, for absenteeism (panel A), and presenteeism (panel B), respectively. Further details are provided in the Supplemental Methods.

Days per month lost to absenteeism and presenteeism were accounted for using the human capital approach, where days lost to presenteeism and days lost to absenteeism were valued equally [51]. The value of one working day (7 h) was monthly salary divided by monthly working days, adjusted for employment rate. The monthly median gross salary in January 2020 was £1,850 according to the UK Office of National Statistics [52]. Based on 20.83 working days per month (250 per year) the cost of a full lost day was £67.75, accounting for the Office of National Statistics 76.3% employment rate in the 18–64 age range [53].

### Testing uncertainty in the model

The accuracy of model outcomes is subject to structural and parameter uncertainty, so a series of standard tests were conducted to characterise the leading contributors. One-way sensitivity analysis (OWSA) was used to test in turn the sensitivity of net monetary benefit (at £20,000 per QALY) to a fixed 10% each-way variation in each input parameter. Probabilistic sensitivity analysis (PSA) was used to test the sensitivity of total costs and QALYs to simultaneous variation of the input parameters. Age, gender, life expectancy and baseline migraine frequency parameters were excluded from probabilistic variation since these were sampled in the creation of unique individuals. In scenario analyses, alternative assumptions explored the structural aspects of the model most open to uncertainty (e.g., duration of the BSC treatment effect, inclusion of natural history, and discontinuation due to other causes, which were not specified in the short follow-up of the DELIVER trial).

## Results

Five-thousand unique individuals were successfully generated in the micro-simulation, creating the TF3+ population of EM and CM subpopulations. Tables 2 and 3 present the main deterministic health and cost findings, discounted at 3.5% per annum, as per standard practice for the UK setting [24]. Undiscounted findings are also given in the text.

**Table 2:** Health outcomes for full and subpopulations in short and long-term time horizons

Mean outcome	TF3+	TF3+ EM only	TF3+ CM only
Baseline attributes			
Age (IQR; SD)	45.4 (37.2, 53.0; 11.48)		
Proportion CM, %	45.9	–	–
MMDs (IQR)	14.3 (9.0, 20.3)	9.2 (8.2, 10.1)	20.3 (17.9,2 2.8)
5-year truncated time horizon^a^			
Proportion transforming, %	15.2	–	15
Age at transformation (yrs) (IQR)	49.5 (46.5, 52.6)	–	49.5
Proportion resolving, %	13.9		
Age at resolution (yrs) (IQR)	52.9 (49.9, 56.0)	52.8	53.0
Days with migraine, eptinezumab	643	426	899
Days with migraine, BSC	789	521	1,105
Years on treatment, eptinezumab	1.6	1.2	1.9
Years on treatment, BSC	0.4	0.3	0.4
Proportion TEAE discontinuation, % (timing, yrs) (SD)	4.3 (0.09)	3.7 (0.08)	5.1 (0.11)
Proportion other-cause discontinuation, % (timing, yrs) (SD)	20.1 (0.20)	16.0 (0.16)	24.9 (0.25)
Proportion super-responding, %	3.2	3.2	3.2
Lifetime horizon			
Proportion transforming, %	62.8	–	62.8
Age at transformation (yrs)	50.6	–	50.6
Proportion resolving, %	73		
Age at resolution (yrs)	54.5	54.4	54.6
Years with migraine from start of treatment, eptinezumab	14.5	13.3	16.0
Years with migraine from start of treatment, BSC	16.0	15.7	16.3
Days with migraine, eptinezumab	2,886	2,198	3,699
Days with migraine, BSC	3,245	2,437	4,199
Years on treatment, eptinezumab	3.6	2.7	4.6
Years on treatment, BSC	0.4	0.3	0.4
Proportion TEAE discontinuation, % (timing, yrs) (SD)	10.7 (0.95)	8.2 (0.71)	13.7 (1.22)
Proportion other-cause discontinuation, % (timing, yrs) (SD)	20.1 (0.20)	16.0 (0.16)	24.9 (0.25)

*Abbreviations BSC* Best supportive care, *CM* Chronic migraine, *EM* Episodic migraine, *IQR* Interquartile range, *MMD* Monthly migraine days, *SD* standard deviation, *TEAE* Treatment-emergent adverse event, *TF3*+ At least three prior preventive treatment failures

Key: ^a^Means were truncated when events remained possible after 5 years

**Table 3:** Economic outcomes for full and subpopulations in short and long-term time horizons

Mean outcome	TF3+	TF3+ EM only	TF3+ CM only
5-year horizon			
QALYs, eptinezumab	2.95	3.17	2.70
QALYs, BSC	2.71	2.99	2.37
Discounted QALYs, eptinezumab	2.72	2.92	2.48
Discounted QALYs, BSC	2.49	2.75	2.17
Cost of eptinezumab acquisition	£7,497	£5,998	£9,267
Cost of eptinezumab administration	£967	£773	£1,195
Cost of disease management, eptinezumab	£2,399	£2,212	£2,621
Cost of disease management, BSC	£2,548	£2,356	£2,775
Cost of rescue therapy, eptinezumab	£780	£517	£1,091
Cost of rescue therapy, BSC	£957	£632	£1,340
Cost of work impairment, eptinezumab	£33,650	£27,425	£41,000
Cost of work impairment, BSC	£47,257	£40,332	£55,433
Total undiscounted cost, eptinezumab	£45,294	£36,925	£55,173
Total undiscounted cost, BSC	£50,762	£43,320	£59,548
Total discounted cost, eptinezumab	£41,804	£34,078	£50,925
Total discounted cost, BSC	£46,698	£39,829	£54,809
Cost-saving versus BSC	£4,894	£5,751	£3,883
ICER, £	Dominant in the SE quadrant	Dominant in the SE quadrant	Dominant in the SE quadrant
Net monetary benefit @ £20,000, £	£9,515	£8,938	£10,010
Lifetime horizon			
QALYs, eptinezumab	22.30	22.95	21.53
QALYs, BSC	21.72	22.54	20.74
Discounted QALYs, eptinezumab	11.91	12.38	11.35
Discounted QALYs, BSC	11.46	12.06	10.75
Cost of eptinezumab acquisition	£18,463	£14,025	£23,702
Cost of eptinezumab administration	£2,380	£1,808	£3,056
Cost of disease management, eptinezumab	£15,006	£14,428	£15,688
Cost of disease management, BSC	£15,406	£14,823	£16,087
Cost of rescue therapy, eptinezumab	£3,500	£2,666	£4,485
Cost of rescue therapy, BSC	£3,936	£2,957	£5,091
Cost of work impairment, eptinezumab	£179,749	£162,457	£200,163
Cost of work impairment, BSC	£258,749	£238,088	£282,824
Total undiscounted cost, eptinezumab	£219,098	£195,384	£247,093
Total undiscounted cost, BSC	£277,943	£255,869	£304,003
Total discounted cost, eptinezumab	£133,127	£115,610	£153,806
Total discounted cost, BSC	£163,225	£147,121	£182,236
ICER, £	Dominant in the SE quadrant	Dominant in the SE quadrant	Dominant in the SE quadrant
Net monetary benefit @ £20,000	£39,056	£37,892	£40,429

*Abbreviations*: *BSC* Best supportive care, *CM* Chronic migraine, *EM* Episodic migraine, *ICER* Incremental cost-effectiveness ratio, *IQR* Interquartile range, *QALY* Quality-adjusted life-year, *SE* South-east, *TF3*+ At least three prior preventive treatment failures

### Typical patient over five years

The average person entering the simulation with EM or CM was female, aged 45 years (range 18–85), lived to 81 years, and began eptinezumab or BSC treatment with 14 MMDs. Five years after first commencing eptinezumab treatment and having spent 19 months on treatment, this typical person will have experienced 146 fewer days with migraine compared to BSC, equivalent to a gain of 0.23 discounted QALYs. In a responder-only comparison, the typical benefit of eptinezumab treatment after 5 years, with 34 months on treatment, was 272 fewer migraine days and a gain of 0.44 undiscounted QALYs. Fifteen percent of all individuals transformed from CM to EM and for 14% migraine was therapeutically resolved, but only in 4% of people did any natural improvement occur whilst on actively using preventive migraine treatment. The risk of discontinuation due to TEAEs was 4% and due to other causes was 20%. The mean times to these events were 1 month and 2.5 months after commencement of treatment, respectively. Three percent of patients treated with eptinezumab experienced a ‘super-response’ in which the treatment effect continued through and beyond the treatment holiday despite discontinuation (also known as ‘positive stopping’).

Over 5 years, the average eptinezumab patient with EM began with 9 MMDs and avoided 95 migraine days (220 if a responder), of which 15 months were spent on treatment, yielding a gain of 0.17 discounted QALYs. The average eptinezumab patient with CM began with 20.3 MMDs and avoided 205 migraine days (306 if a responder), of which 23 months were spent on treatment, yielding a gain of 0.31 discounted QALYs.

For the NHS payer, the cost of eptinezumab for the typical patient was £7,497 over 5 years, plus £967 for its administration. These are partly offset by fewer days with migraine, leading to savings of £149 from less intense disease management, and £177 from reduced need for rescue therapy. For the societal payer, improved work productivity led to an economic gain to society of £13,607 over 5 years, so that overall, after discounting both future costs and benefits, the typical patient gained 0.23 QALYs against a net saving of £4,894. This represents health benefit versus BSC that is gained with a saving, and in cost-effectiveness terms, eptinezumab is dominant over BSC. When accounting for direct healthcare resource costs only, eptinezumab has net cost of £7,655 for the same benefit, producing an ICER of £33,138 per QALY gained.

### Analysis of response threshold in chronic migraine

When the response threshold in CM is increased from ≥ 30% to ≥ 50% the mean MMD treatment benefit in responders is improved as the proportion responding is decreased, with the opposite effect for non-responders. The net effect was an average of four fewer months on treatment, more migraine days, and 0.05 fewer QALYs gained.

### One-way sensitivity analysis

**Fig. 4:**
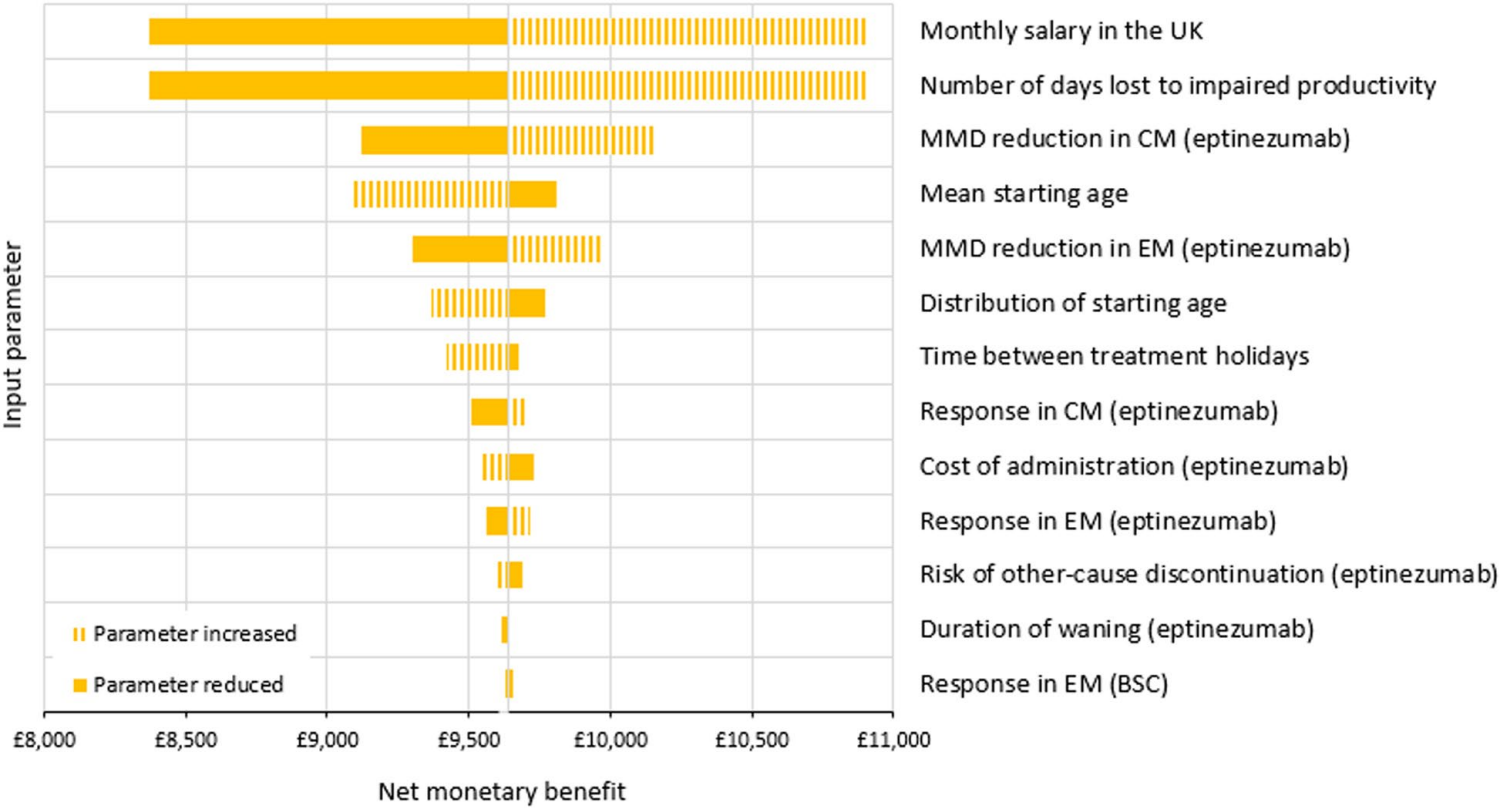
A tornado plot of key parameters and their impact on net monetary benefit (at £20,000 per QALY) when varied. Abbreviations: BSC, best supportive care; EM, episodic migraine; CM, chronic migraine; UK, United Kingdom

OWSA was used to identify those parameters for which the net monetary benefit was most sensitive to 10% variation. Figure 4 displays the top 13 results for the TF3+ population. The two parameters informing productivity gain—monthly salary and the number of days lost to impaired productivity—have the largest impact on net monetary benefit; this is the element of cost most improved by eptinezumab. The second most influential were the parameters for MMD reduction, followed by starting age parameters (since a younger entry age increases the economic productivity opportunity).

### Scenario analysis

Alternative assumptions were explored through scenario analyses. Results are presented in Table 4 for the TF3+ population using a lifetime horizon. Net monetary benefit was robust to most alternative positions, including the exclusion of natural history, but showed a reduction from £9,515 to £5,036 when work impairment from presenteeism relative to absenteeism was changed from equivalence to half. To provide context to the overall evaluation, we also examined cost-effectiveness when the analysis perspective was changed from societal payer to health system payer (i.e., NHS). Net monetary benefit was reduced to –£3,035, producing an ICER of £33,138 per QALY.

**Table 4:** Outcomes under alternative assumptions for the TF3+ population

Alternative assumption	Migraines averted versus BSC	Cost of eptinezumab	Incremental discounted total costs	Incremental discounted QALYs	Net monetary benefit
Base case	146	£7,497	–£4,894	0.231	£9,515
Period of BSC benefit doubled to two years	143	£7,497	–£4,850	0.228	£9,411
BSC excluded as a further treatment after a regimen of eptinezumab	141	£7,497	–£4,572	0.226	£9,100
Exclusion of treatment holidays	156	£9,318	–£3,215	0.255	£8,323
Exclusion of natural history and treatment holidays	169	£9,717	–£3,207	0.271	£8,634
Response threshold in chronic migraine raised from 30 to 50% improvement	113	£5,528	–£6,159	0.181	£9,782
Discontinuation from other causes allowed beyond two years	148	£7,431	–£5,059	0.235	£9,759
Disutility included for possible fear/pain/anxiety associated with intravenous administration	146	£7,497	–£4,894	0.204	£8,969
Cost of one day of presenteeism reduced from a full day to half a day	146	£7,497	–£415	0.231	£5,036
One fully impaired day of work productivity for each occasion of eptinezumab administration	146	£7,497	–£4,627	0.231	£9,247
Payer perspective only, exclusion of work productivity benefit	146	£7,497	£7,655	0.231	–£3,035 (ICER = £33,138)

*Abbreviations*: *BSC* Best supportive care, *QALY* Quality-adjusted life-year

### Probabilistic sensitivity analysis

The total cost and total QALY outcomes of the PSA were well matched against the deterministic equivalents for both strategies (Table 5). Probabilistic costs were marginally lower and QALYs were marginally higher than deterministic equivalents. Since the trend was common to both strategies, the PSA ICER was close to the deterministic ICER. This finding and the balanced outputs of the OWSA suggest the absence of non-linearity in parameter behaviour. An analysis of cost-effectiveness acceptability examining the probability of cost-effectiveness up to a willingness-to-pay threshold of £80,000 per QALY showed that eptinezumab was the most cost-effective option in 100% of iterations for thresholds upward of £6,000 per QALY and was dominant in 96% of the iterations.

**Table 5:** Comparison of probabilistic and deterministic outcomes over a 5-year horizon

Mean outcome	Probabilistic [95% CrI]	Deterministic
Total discounted cost, eptinezumab	£41,270 [£26,272: £57,560]	£41,804
Total discounted cost, BSC	£46,620 [£26,087: £68,770]	£46,698
Discounted QALYs, eptinezumab	2.70 [2.43: 2.98]	2.72
Discounted QALYs, BSC	2.46 [2.07: 2.84]	2.49
Incremental costs	–£5,350 [–£11,647: £503]	–£4,894
Incremental QALYs	0.237 [0.071: 0.436]	0.231
ICER	Dominant in the SE quadrant	Dominant in the SE quadrant
Net monetary benefit @ £20,000	£10,085	£9,515

*Abbreviations*: *BSC* Best supportive care, *CrI* Credible interval, *ICER* Incremental cost-effectiveness ratio, *QALY* Quality-adjusted life-year, *SE* South-east

**Fig. 5:**
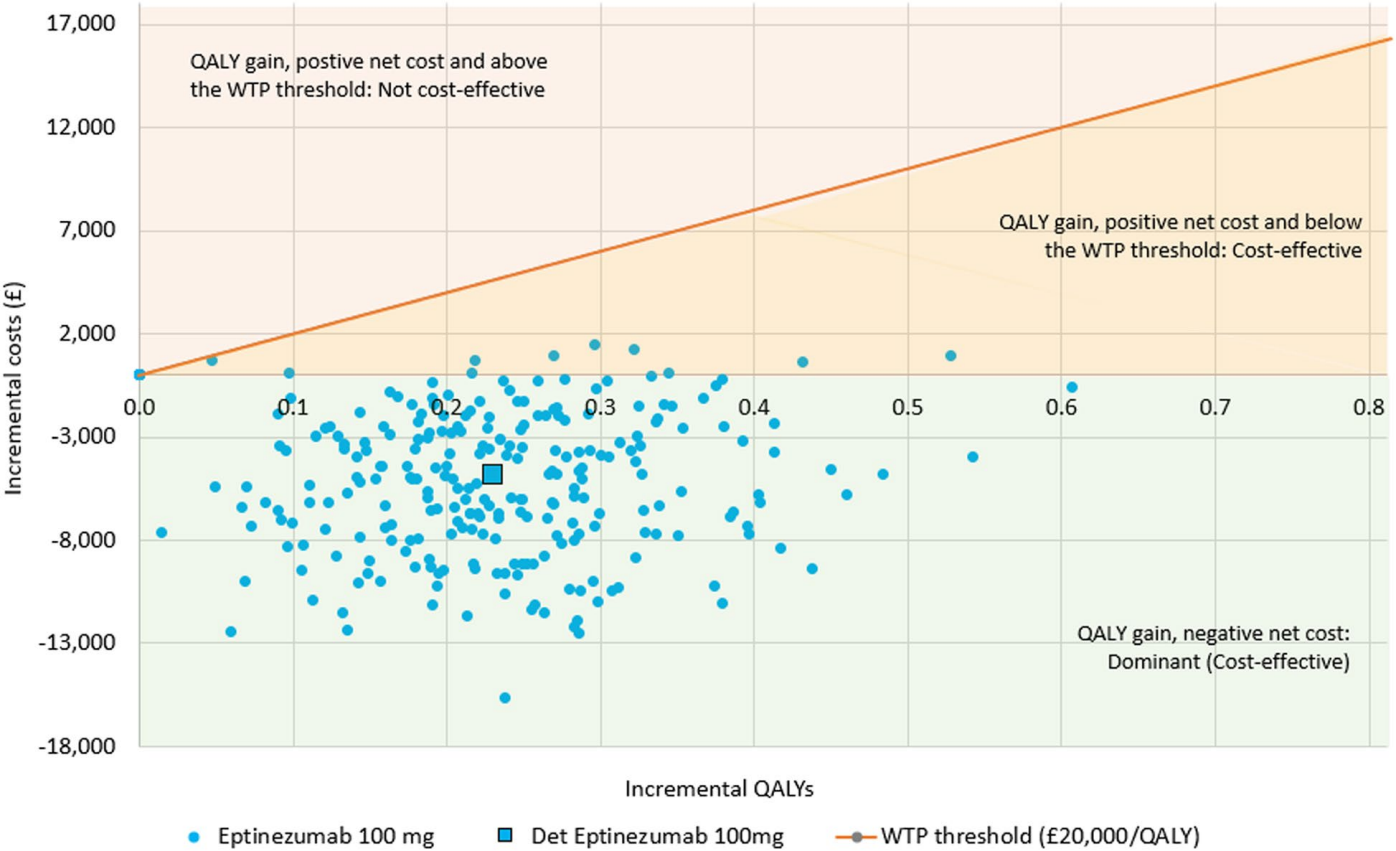
Probabilistic outcomes on the cost-effectiveness plane, 5-year time horizon. Note on figure: Only the Northeast and Southeast quadrants of the cost-effectiveness plane are presented since no probabilistic sensitivity analysis iterations produced negative incremental QALYs. Abbreviations: Det, Deterministic; QALY, Quality-adjusted life-year; WTP, willingness-to-pay

A plot of the 250 probabilistic iterations on the cost-effectiveness plane (Fig. 5) depicts a dispersion centred around the deterministic mean, with slightly lower variation on the incremental cost axis than the incremental QALY.

### Validation, calibration, and quality control

Averaged sampled baseline characteristics in the model closely matched the intended population. The proportion of patients who were female was 89.1% in the model versus 88.7% in the TF3+ cohort of DELIVER. Mean age at entry/treatment commencement was 45.4 years (model) versus 45.2 years (DELIVER TF3+ cohort) and mean MMDs at model entry was 14.3 MMDs (model) versus 14.3 MMDs (DELIVER TF3+ cohort). The proportion with CM at entry was 45.9% in the model versus 45.6% in the real-world source, and this was comparable to 49.1% in the TF3+ cohort of DELIVER.

Average time on treatment (eptinezumab) was calibrated to the most appropriate long-term estimates. To model the time on treatment beyond the short follow-up of the DELIVER trial, the combined rate of discontinuation in the model using a lifetime horizon was tuned to a net rate from real-world use of subcutaneous anti-CGRP mAbs (data on file). The resultant model mean was 3.6 years, the distribution positively skewed by the 12-week stopping rule, giving a median of 0.31 years. This was comparable to an estimated mean of 3.0 years and median of 0.29 years from the real-world source.

The model structure and coding were checked in a quality control step by third-party experts and outcomes were reviewed by three clinical experts at early and late stages of development.

## Discussion

This analysis presents the first economic evaluation of eptinezumab from a societal perspective in a UK NHS setting [54]. Additionally, it is the first economic evaluation for a preventive migraine treatment using a discrete event simulation that includes the natural history of migraine.

The QALY analysis found that an eptinezumab strategy produced a gain of 0.23 discounted QALYs over BSC (i.e., no preventive treatment) over a 5-year time horizon for people who have tried three or more other preventive treatments that failed. This compares to erenumab, a subcutaneous anti-CGRP mAb, for which 0.1 QALYs gained versus no preventive treatment was reported in a US analysis over a 2-year time horizon [55], and 0.31 QALYs gained versus BSC in a Swedish analysis over a 10-year time horizon [56]. The economic analysis reported here found that eptinezumab was dominant over BSC, since the QALY gain was associated with a net saving. This five-year result was consistent with the lifetime result in the TF3+ population, as well as in the analysis of EM-only and CM-only subpopulations using either horizon. Scenario analyses showed results were robust to structural changes in the model. Sensitivity analyses testing fixed variation in individual input parameters found the extent and cost of work impairment were most influential, consistent with the NHS payer setting scenario which excluded productivity loss and produced a net cost. PSA and deterministic totals were well aligned, suggesting the absence of potentially distorting non-linearity in input behaviour. All PSA iterations produced ICERs below the £20,000 per QALY, the threshold for cost-effectiveness routinely used by NICE, and in 96% of probabilistic iterations found eptinezumab was dominant over BSC [24].

Similar publicly reported analyses of this decision problem are partly consistent with the findings of this analysis. In the US setting with a 2-year time horizon, an economic evaluation of the subcutaneous anti-CGRP mAb erenumab, which also took the societal perspective, found erenumab was dominant in CM and likely not cost-effective in EM [55]. The $122,167 ICER was equivalent to £86,296 in 2022 [57]. An analysis of the same anti-CGRP mAb in Sweden which used a 10-year horizon returned the same result, although borderline cost-effective in the combined EM/CM population [56]. The main contrast in these comparisons is the result for anti-CGRP mAb candidates who have the more debilitating chronic definition of migraine frequency (i.e., CM).

The absence of natural history modelling has been a key criticism from NICE in previous technology appraisals of preventive migraine treatments [20–22, 58]. There were two main components of natural history model to consider in assessment of its overall impact. First, a series of regular treatment holidays were installed, during which no treatment cost was accrued yet some residual benefit was retained as treatment effect waned. Secondly, natural resolution of migraine led to treatment discontinuation and both transformation and resolution led to utility improvement. However, a pause in anti-CGRP mAb treatment after 12 to 18 months is recommended in European guidelines, which is consistent with this analysis [32]. Further, we found that when accounting for a broad range in treatment age, natural improvements occurred during active treatment period for only a small minority. Considering these potentially overlooked mitigating factors, the role in natural history is unlikely to significantly influence estimates of cost-effectiveness.

Another contention of previous modelling [20–22] is limiting treatment effect to days with migraine when in fact the burden of migraine also depends on individual migraine characteristics [44, 45, 50]. Further support for refinement of approach is evidenced in analysis of patient-identified most bothersome symptom measure in DELIVER [45]. The inclusion here of self-reported health-related quality-of-life benefit arising from preventive treatment, as set against BSC, may partly address the unmet need.

Anti-CGRP mAb stopping rules are not universal. The rule adopted here reflects UK clinical practice and NICE guidance [20–23, 32], but secondary analysis explored a ≥ 50% MMD reduction rule for the CM subpopulation, which was a secondary endpoint of DELIVER [17]. It was found that this more stringent threshold decreased the mean time on treatment, resulting in more migraine days overall and a small reduction in mean QALY gain. However, the stopping rule at 12-weeks does not allow for latent positive responses as were observed in DELIVER at 24 weeks. Many participants in the eptinezumab arm of DELIVER who attained the ≥ 30% but not ≥ 50% improvement over weeks 1–12 went on to attain ≥ 50% reduction over weeks 13–24 [59, 60].

Societal benefit is not routinely considered by regulators. NICE in England prefers the health system (NHS) payer perspective; however, new commissioning preferences are emerging. In the UK, new Integrated Care Systems attempt to connect with wider determinants of health such as education and employment when commissioning healthcare services [61]. The societal perspective adopted by this study is attuned to this shift, since reduced work impairment consequent to treatment with eptinezumab is evidenced, quantified, and meaningful [18].

### Limitations

As for all economic evaluations, there are modelling limitations which should be considered in context to the findings. Firstly, no evidence exists yet to inform long-term safety or benefit of treatment. Informed predictions are made for durability of response, treatment-emergent adverse events, and time on treatment. Secondly, a central assumption is that the treatment effect of BSC in the model approximates to the placebo arm of DELIVER, a study in which concomitant preventive treatment was not permitted. The in-trial treatment effect of placebo may overestimate mean peak effect in the real-world because participants had significant treatment history i.e., they had already tried and failed at least three preventive therapies. Nor would patients in the real-world attending clinic receive a placebo infusion and the associated contextual affects that go with it. Conversely, real-world BSC may include newly arriving experimental medical interventions, the impact of which would not be observed in a trial setting. Thirdly, the incomplete characterisation of natural history in the literature, at least for modelling purposes, has required some generalising assumptions, each with a degree of uncertainty. Fourthly, the analysis is based on publicly available prices. Additional confidential discounts to the NHS as part of Patient Access Schemes are not included. Consequently, the presented economic results are likely conservative. Finally, this study does not explore the impact of migraine prevention on longer-term educational opportunities and career trajectory. 

## Conclusions

The reduction in the burden of migraine with eptinezumab treatment relative to BSC, as captured in the DELIVER clinical trial, equated to a meaningful QALY gain consistent with subcutaneously administered anti-CGRP mAbs. From a societal perspective in which productivity impairment is considered, eptinezumab is cost-saving compared to BSC; therefore, eptinezumab is a cost-effective treatment for people with at least 4 migraine days per month who have tried three or more other preventive treatments that have failed.

### Supplementary Information

**Supplementary Material 1.**

## Data Availability

All data generated or analysed during this study are included in this published article (and its supplementary content).

## Abbreviations

Anti-CGRP mAb: Anti-calcitonin gene-related peptide monoclonal antibody
BSC: Best supportive care
CM: Chronic migraine
EM: Episodic migraine
ICER: Incremental cost-effectiveness ratio
IV: Intravenously
MHD: Monthly headache day
MMD: Monthly migraine day
MSQ: Migraine-Specific Quality of life questionnaire
NHS: National Health Service
NICE: National Institute for Health and Care Excellence
OWSA: One-way sensitivity analysis
PSA: Probabilistic sensitivity analysis
QALYs: Quality-adjusted life-years
TEAEs: Treatment-emergent adverse events
TF3+ population: Population with at least three prior preventive migraine treatment failures
UK: United Kingdom
WPAI: Work Productivity and Activity Impairment

## References

[1] Headache Classification Committee of the International Headache Society (IHS) (2018). The international classification of headache disorders, 3rd edition. Cephalalgia.

[2] GBD 2019 Diseases and Injuries Collaborators (2020). Global burden of 369 diseases and injuries in 204 countries and territories, 1990–2019: a systematic analysis for the global burden of disease study 2019. Lancet.

[3] Institute for Health Metrics and Evaluation (2019) GBD Compare https://vizhub.healthdata.org/gbd-compare/. Accessed 17 Apr 2023

[4] Affatato O, Miguet M, Schiöth HB, Mwinyi J (2021). Major sex differences in migraine prevalence among occupational categories: a cross-sectional study using UK Biobank. J Headache Pain.

[5] Steiner TJ, Scher AI, Stewart WF, Kolodner K, Liberman J, Lipton RB (2003). The prevalence and disability burden of adult migraine in England and their relationships to age, gender and ethnicity. Cephalalgia.

[6] Buse D, Manack A, Serrano D, Reed M, Varon S, Turkel C, Lipton R (2012). Headache impact of chronic and episodic migraine: results from the American migraine prevalence and prevention study. Headache.

[7] Buse DC, Reed ML, Fanning KM, Bostic R, Dodick DW, Schwedt TJ (2020). Comorbid and co-occurring conditions in migraine and associated risk of increasing headache pain intensity and headache frequency: results of the migraine in America symptoms and treatment (MAST) study. J Headache Pain.

[8] Buse DC, Reed ML, Fanning KM, Bostic RC, Lipton RB (2020). Demographics, headache features, and comorbidity profiles in relation to headache frequency in people with migraine: results of the American Migraine Prevalence and Prevention (AMPP) study. Headache.

[9] Munjal S, Singh P, Reed ML, Fanning K, Schwedt TJ, Dodick DW (2020). Most bothersome symptom in persons with migraine: results from the Migraine in America symptoms and treatment (MAST) study. Headache.

[10] Walters AB, Hamer JD, Smitherman TA (2014). Sleep disturbance and affective comorbidity among episodic migraineurs. Headache.

[11] Brown JS, Neumann PJ, Papadopoulos G, Ruoff G, Diamond M, Menzin J (2008). Migraine frequency and health utilities: findings from a multisite survey. Value Health.

[12] Buse DC, Fanning KM, Reed ML, Murray S, Dumas PK, Adams AM, Lipton RB (2019). Life with migraine: effects on relationships, career, and finances from the chronic migraine epidemiology and outcomes (CaMEO) study. Headache.

[13] Lampl C, Thomas H, Stovner LJ, Tassorelli C, Katsarava Z, Laínez JM (2016). Interictal burden attributable to episodic headache: findings from the eurolight project. J Headache Pain.

[14] The Work Foundation (2018) Society’s headache. The socioeconomic impact of migraine. https://www.lancaster.ac.uk/media/lancaster-university/content-assets/documents/lums/work-foundation/SocietysHeadacheTheSocioeconomicimpactofmigraine.pdf. Accessed 17 Apr 2023.

[15] Ford J, Nichols RM, Ye W, Tockhorn-Heidenreich A, Cotton S, Jackson J (2021). Patient-reported outcomes for migraine in the US and Europe: burden Associated with multiple preventive treatment failures. Clinico econ Outc Res..

[16] Goadsby PJ, Evers S (2020). International classification of Headache disorders - ICHD-4 alpha. Cephalalgia.

[17] Ashina M, Lanteri-Minet M, Pozo-Rosich P, Ettrup A, Christoffersen CL, Josiassen MK (2022). Safety and efficacy of eptinezumab for migraine prevention in patients with two-to-four previous preventive treatment failures (DELIVER): a multi-arm, randomised, double-blind, placebo-controlled, phase 3b trial. Lancet Neurol.

[18] Barbanti P, Goadsby PJ, Lambru G, Ettrup A, Christoffersen CL, Josiassen MK (2022). Effects of eptinezumab on self-reported work productivity in adults with migraine and prior preventive treatment failure in the randomized, double-blind, placebo-controlled DELIVER study. J Headache Pain.

[19] EMC, Summary of product characteristics (2023) VYEPTI 100 mg concentrate for solution for infusion. https://www.medicines.org.uk/emc/product/13243/smpc. Accessed 17 Apr 2023

[20] National Institute for Health and Care Excellence (NICE) (2020) TA659: Galcanezumab for preventing migraine. https://www.nice.org.uk/guidance/TA659. Accessed 17 Apr 2023 40875874

[21] National Institute for Health and Care Excellence (NICE) (2021) TA682: Erenumab for preventing migraine. https://www.nice.org.uk/guidance/ta682. Accessed 17 Apr 2023 40737473

[22] National Institute for Health and Care Excellence (NICE) (2022) TA764: Fremanezumab for preventing migraine. https://www.nice.org.uk/guidance/TA764. Accessed 17 Apr 2023 40455896

[23] National Institute for Health and Care Excellence (NICE) (2023) TA871: Eptinezumab for preventing migraine. https://www.nice.org.uk/guidance/ta871/. Accessed 17 Apr 2023 40080618

[24] National Institute for Health and Care Excellence (NICE) (2022) NICE health technology evaluations: the manual. https://www.nice.org.uk/process/pmg36/. Accessed 17 April 2023

[25] NHS England (2022) What are integrated care systems? https://www.england.nhs.uk/integratedcare/what-is-integrated-care/. Accessed 8 May 2023

[26] The King’s Fund (2022) Integrated care systems explained: making sense of systems, places and neighbourhoods. https://www.kingsfund.org.uk/publications/integrated-care-systems-explained. Accessed 8 May 2023

[27] Kernick D, Kondori N, Pain A, Mount J, Appel C, Ranopa M, Gulati T (2024). Preventive treatment patterns in the adult migraine population: an observational UK study over 7 years. BMC Primary Care.

[28] Office for National Statistics (ONS) (2021) National Life Tables: England. https://www.ons.gov.uk/peoplepopulationandcommunity/birthsdeathsandmarriages/lifeexpectancies/bulletins/nationallifetablesunitedkingdom/2018to2020. Accessed 17 Apr 2023

[29] Karnon J, Haji Ali Afzali H (2014). When to use discrete event simulation (DES) for the economic evaluation of health technologies? A review and critique of the costs and benefits of DES. Pharmacoeconomics.

[30] Ailani J, Burch RC, Robbins MS (2021). The American headache society consensus statement: update on integrating new migraine treatments into clinical practice. Headache.

[31] Raffaelli B, Terhart M, Overeem LH, Mecklenburg J, Neeb L, Steinicke M, Reuter U (2022). Migraine evolution after the cessation of CGRP(-receptor) antibody prophylaxis: a prospective, longitudinal cohort study. Cephalalgia.

[32] Sacco S, Amin FM, Ashina M, Bendtsen L, Deligianni CI, Gil-Gouveia R (2022). European Headache Federation guideline on the use of monoclonal antibodies targeting the calcitonin gene related peptide pathway for migraine prevention – 2022 update. J Headache Pain.

[33] Manack A, Buse DC, Serrano D, Turkel CC, Lipton RB (2011). Rates, predictors, and consequences of remission from chronic migraine to episodic migraine. Neurology.

[34] Roy J, Stewart WF (2011). Methods for estimating remission rates from cross-sectional survey data: application and validation using data from a national migraine study. Am J Epidemiol.

[35] Serrano D, Lipton RB, Scher AI, Reed ML, Stewart WF, Adams AM, Buse DC (2017). Fluctuations in episodic and chronic migraine status over the course of 1 year: implications for diagnosis, treatment and clinical trial design. J Headache Pain.

[36] Krause DN, Warfvinge K, Haanes KA, Edvinsson L (2021). Hormonal influences in migraine — interactions of oestrogen, oxytocin and CGRP. Nat Reviews Neurol.

[37] Lipton RB, Bigal ME, Diamond M, Freitag F, Reed ML, Stewart WF, Group obotAA (2007). Migraine prevalence, disease burden, and the need for preventive therapy. Neurology.

[38] Pokoradi AJ, Iversen L, Hannaford PC (2011). Factors associated with age of onset and type of menopause in a cohort of UK women. Am J Obstet Gynecol.

[39] Paramsothy P, Harlow SD, Nan B, Greendale GA, Santoro N, Crawford SL (2017). Duration of the menopausal transition is longer in women with young age at onset: the multiethnic study of women’s Health across the Nation. Menopause.

[40] Kudrow D, Cady RK, Allan B, Pederson SM, Hirman J, Mehta LR, Schaeffler BA (2021). Long-term safety and tolerability of eptinezumab in patients with chronic migraine: a 2-year, open-label, phase 3 trial. BMC Neurol.

[41] GSK (1998) Migraine-Specific Quality-of-Life Questionannaire (MSQ Version 2.1). https://eprovide.mapi-trust.org/instruments/migraine-specific-quality-of-life-questionnaire. Accessed July 2023

[42] Gillard PJ, Devine B, Varon SF, Liu L, Sullivan SD (2012). Mapping from disease-specific measures to health-state utility values in individuals with migraine. Value Health.

[43] EuroQol (2019) EQ-5D-5L User Guide. https://euroqol.org/publications/user-guides/. Accessed July 2023 2023

[44] Jonsson L, Regnier SA, Kymes S, Awad SF, Talon B, Lee XY, Goadsby PJ (2023). Estimating treatment effects on health utility scores for patients living with migraine: a post hoc analysis of the DELIVER trial. Expert Rev Pharmacoecon Outcomes Res.

[45] Goadsby PJ, Barbanti P, Lambru G, Ettrup A, Christoffersen CL, Josiassen MK (2023). Eptinezumab improved patient-reported outcomes and quality of life in patients with migraine and prior preventive treatment failures. Eur J Neurol.

[46] NICE (2022) British National Formulary (BNF). https://bnf.nice.org.uk. Accessed 17 Apr 2023

[47] National Institute for Health and Care Excellence (NICE) (2010) Adalimumab, etanercept, infliximab, rituximab and abatacept for the treatment of rheumatoid arthritis after the failure of a TNF inhibitor. https://www.nice.org.uk/guidance/ta195. Accessed 7 July 2023

[48] NHS England. 2019/20 National Cost Collection Data Publication 2021 [updated 27 July 2022]. Available from: https://www.england.nhs.uk/publication/2019-20-national-cost-collection-data-publication/. Accessed 17 Apr 2023

[49] Curtis LAB (2020). Amanda. Unit Costs of Health & Social Care 2020.

[50] Barbanti P, Awad SF, Regnier SA, Lee X, Goadsby PJ (2023). Impact of eptinezumab on work productivity beyond reductions in monthly migraine days: Post hoc analysis of the DELIVER trial (P13-12.012). Neurology.

[51] Linde M, Gustavsson A, Stovner LJ, Steiner TJ, Barré J, Katsarava Z (2012). The cost of headache disorders in Europe: the Eurolight project. Eur J Neurol.

[52] Office for National Statistics (ONS) (2022) Earnings and employment from Pay As You Earn Real Time Information, UK: June 2022. https://www.ons.gov.uk/employmentandlabourmarket/peopleinwork/earningsandworkinghours/bulletins/earningsandemploymentfrompayasyouearnrealtimeinformationuk/june2022. Accessed 7 July 2023.

[53] Office for National Statistics (ONS): Labour market overview, UK (2020) https://www.ons.gov.uk/employmentandlabourmarket/peopleinwork/employmentandemployeetypes/bulletins/uklabourmarket/january2020. Accessed 7 July 2023

[54] Khanal S, Underwood M, Naghdi S, Brown A, Duncan C, Matharu M, Mistry H (2022). A systematic review of economic evaluations of pharmacological treatments for adults with chronic migraine. J Headache Pain.

[55] Sussman M, Benner J, Neumann P, Menzin J (2018). Cost-effectiveness analysis of erenumab for the preventive treatment of episodic and chronic migraine: results from the US societal and payer perspectives. Cephalalgia.

[56] Mahon R, Lang A, Vo P, Huels J, Cooney P, Danyliv A (2021). Cost-effectiveness of erenumab for the preventive treatment of migraine in patients with prior treatment failures in Sweden. Pharmacoeconomics.

[57] Shemilt I, James T, Marcello M (2010). A web-based tool for adjusting costs to a specific target currency and price year. Evid Policy.

[58] National Institute for Health and Care Excellence (NICE) (2012) TA260: Botulinum toxin type A for the prevention of headaches in adults with chronic migraine. https://www.nice.org.uk/guidance/TA260. Accessed 17 Apr 2023

[59] Lipton RB, Silberstein SD (2015). Episodic and chronic migraine headache: breaking down barriers to optimal treatment and prevention. Headache.

[60] Dodick DW, Silberstein SD, Bigal ME, Yeung PP, Goadsby PJ, Blankenbiller T (2018). Effect of Fremanezumab compared with placebo for prevention of episodic migraine: a randomized clinical trial. JAMA.

[61] Fund (2020) TKs: Integrated care systems explained: making sense of systems, places and neighbourhoods. https://www.kingsfund.org.uk/publications/integrated-care-systems-explained. Accessed

