# Supplementary material for: An economic evaluation of eptinezumab for the preventive treatment of migraine in the UK, with consideration for natural history and work productivity
Source: J Headache Pain. 2024 Apr 18;25(1):59. doi: 10.1186/s10194-024-01749-8 (PMC11027549; doi:10.1186/s10194-024-01749-8)
Supplement: Supplementary file 1 — Supplementary Material 1. [file 10194_2024_1749_MOESM1_ESM.pdf]

Supplemental Methods

Additional details of methods and input calculation are provided in this section.

Age at entry

The cumulative distribution function was constructed from TF3+ participants of DELIVER (all arms). This is depicted by the grey line and shown for comparison against a real-world source (orange and blue lines) (**Figure S1**). Age at entry was assumed to follow a normal distribution around the mean (SD) age of 45.2 (11.8) years. Mean (SD) age from the real-world source was similar at 46.5 (12.3) years.

Figure S1. Normal distribution for sampling age at model entry / commencement of treatment

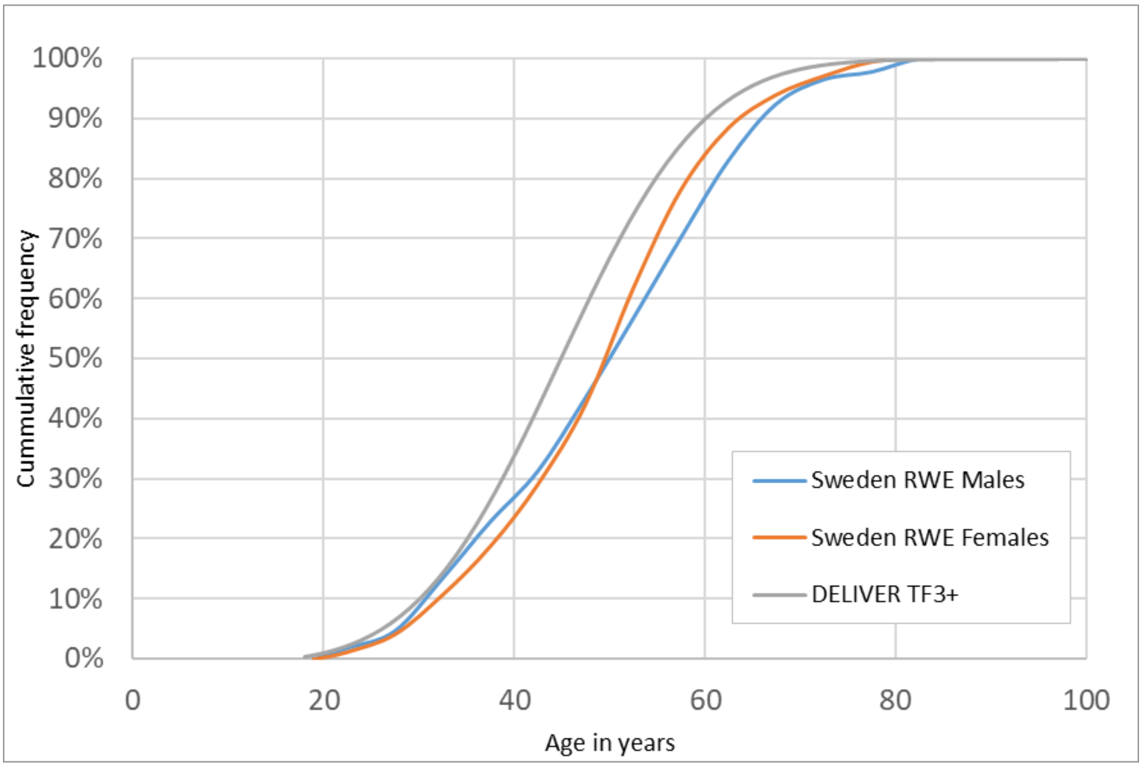

RWE, Real-world evidence; TF3+, At least three prior preventive treatment failures.

Life expectancy

Gompertz distributions were fitted to recent Office for National Statistics data for life expectancy by gender (**Figure S2**). Simulated individuals entered the model with pre-specified age of death; this was independent of other baseline characteristics besides gender.

Figure S2. Gompertz distributions for sampling of life expectancy

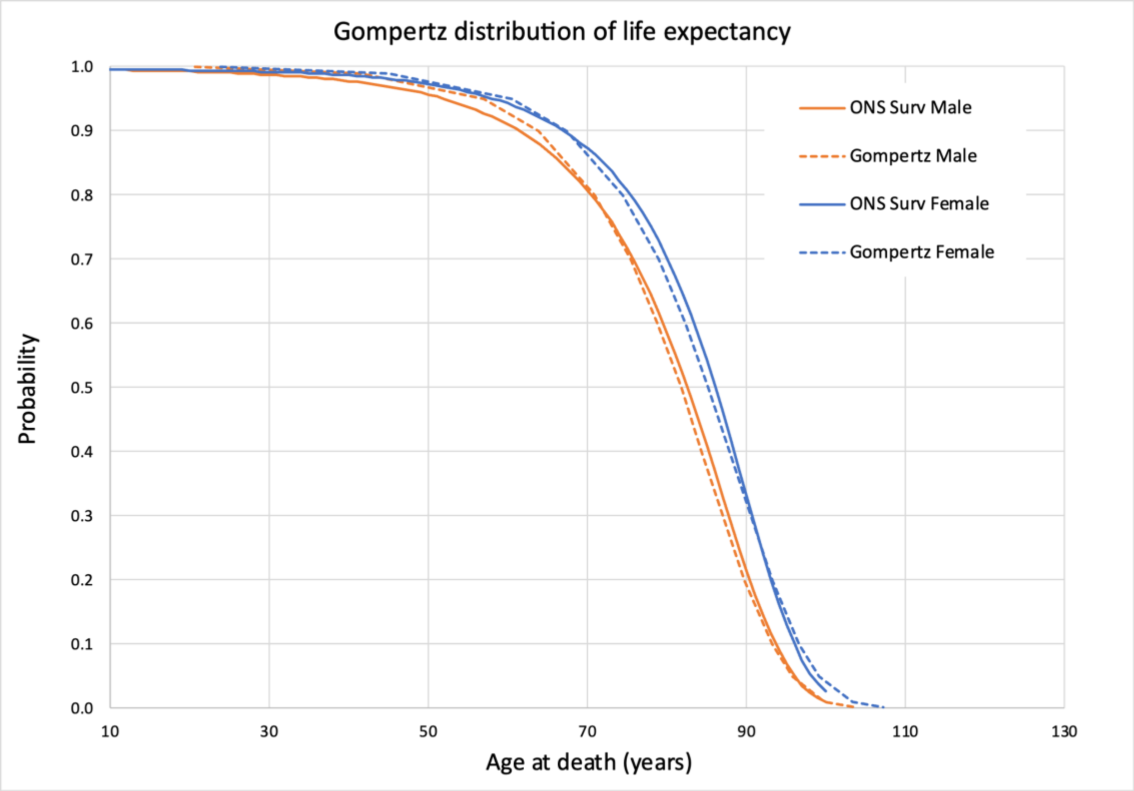

ONS Surv, Office of National Statistics survey.

MMDs at baseline

After simulated individuals were assigned to either EM or CM status, their MMD frequency was sampled. Beta distributions were fitted to data from the respective subpopulations of the TF3+ cohort of the DELIVER trial (dark blue and grey) (**Figure S3**). Mean (SD) MMDs were 9.8 (2.5) for EM and 19.9 (4.0) for CM. A beta distribution was also fitted to the composite data (yellow) and shown for reference next to the naive data (light blue). The dashed orange curve shows the larger TF3+ cohort. Sampling of baseline MMDs was independent of age and gender.

Figure S3. Beta distributions for sampling MMDs at model entry

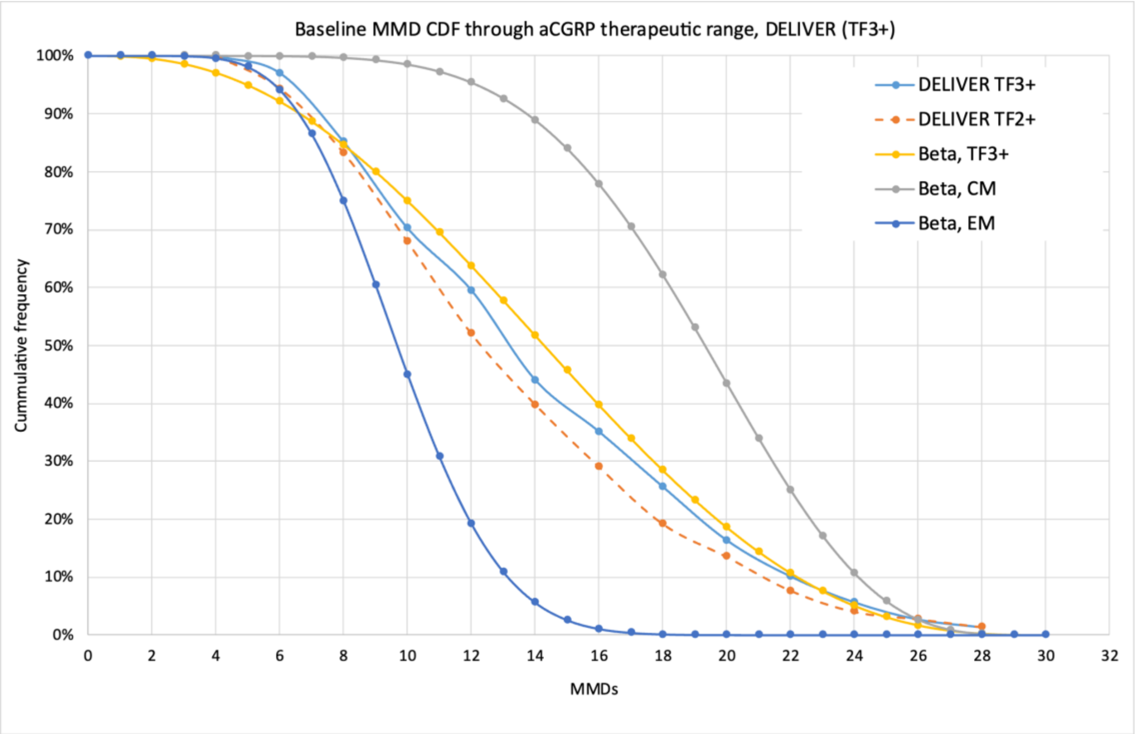

aCGRP, anti-calcitonin gene-related peptide; CDF, cumulative distribution function; CM, chronic migraine; EM, episodic migraine; MMD(s), monthly migraine day(s); TF2+, At least two prior preventive treatment failures TF3+, At least three prior preventive treatment failures.

Other-cause discontinuation

Real-world evidence from subcutaneous anti-CGRP mAbs in people with chronic migraine in Sweden indicated that simple extrapolation of the short-term in-trial rate of adverse event discontinuation in DELIVER would likely underestimate the two-year rate of discontinuation for any reason.[1] Since reasons for discontinuation in the longer-run are likely broader than adverse events alone, an 'other-cause' rate was introduced into the model. This provided a means of calibrating the overall discontinuation rate to match the real-world source, as interpreted by expert clinical opinion, which was a diminishing risk up to two years of treatment (**Figure S4**). The determined exponential rate through years one and two was 0.3.

Figure S4. Exponential sampling distribution of time to other-cause discontinuation

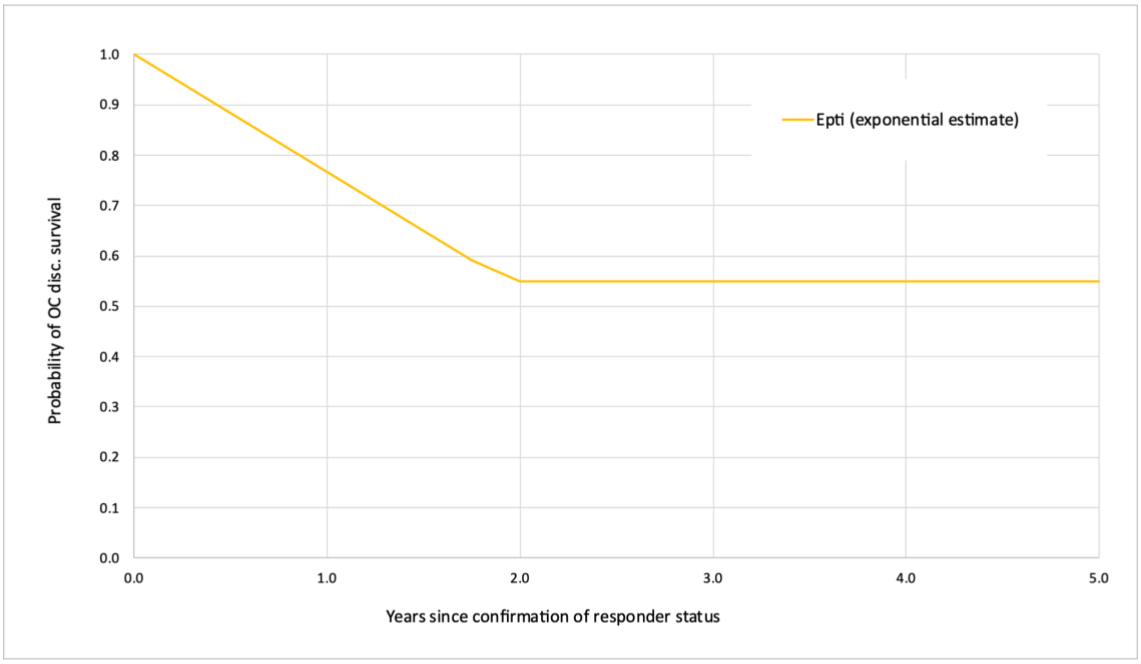

[1] K. Olsson and colleagues, Lundbeck Nordic Market Access, 2022. Unpublished.

Discontinuation due to treatment emergent adverse events

All individuals were at risk of a TEAE leading to discontinuation during the eptinezumab assessment period, and Responders continued to be at risk thereafter. The rate of these event after assessment was based on the two-year open-label PREVAIL trial.[2] The observed rate was extrapolated using an exponential parametric function (**Figure S5**). The exponent of 0.03 was based on the 5.5% withdrawal rate at the end of the open-label study (2 years).

Figure S5. Exponential sampling distribution of time to treatment-emergent adverse event (TEAE) discontinuation

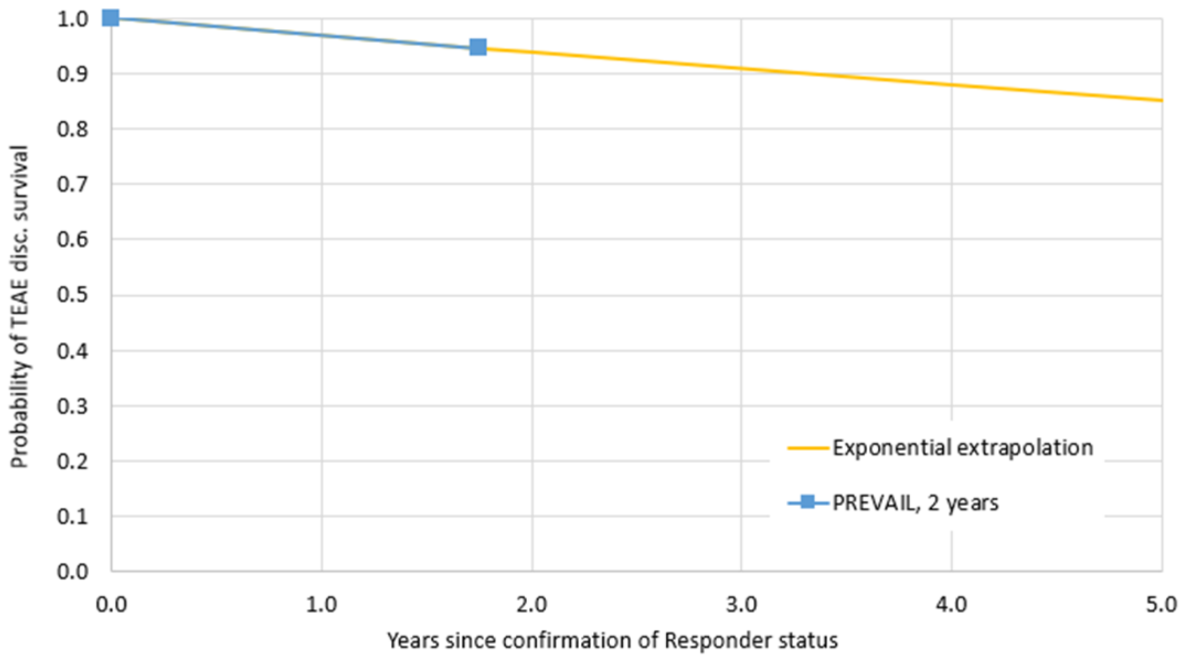

[2] Kudrow D, Cady RK, Allan B, et al. Long-term safety and tolerability of eptinezumab in patients with chronic migraine: a 2-year, open-label, phase 3 trial. BMC Neurol. 2021 Mar 19;21(1):126. doi: 10.1186/s12883-021-02123-w.

Natural history of migraine

Improvement in natural history was linked to age at established menopause (perimenopause may be linked with higher migraine frequency).[3] Since 89% of modelled individuals were women, this concept was applied to all individuals and implemented through transformation (CM to EM) and resolution events (EM to residual level migraine frequency) (**Figure S6**). Age at transformation was randomly sampled from a normal distribution of mean (SD) age 49.5 (5.0) years.[4] Resolution followed a fixed period equating to the mean duration of menopause symptoms (4.3 years).

Figure S6. Age at menopause for sampling of age at transformation from CM to EM

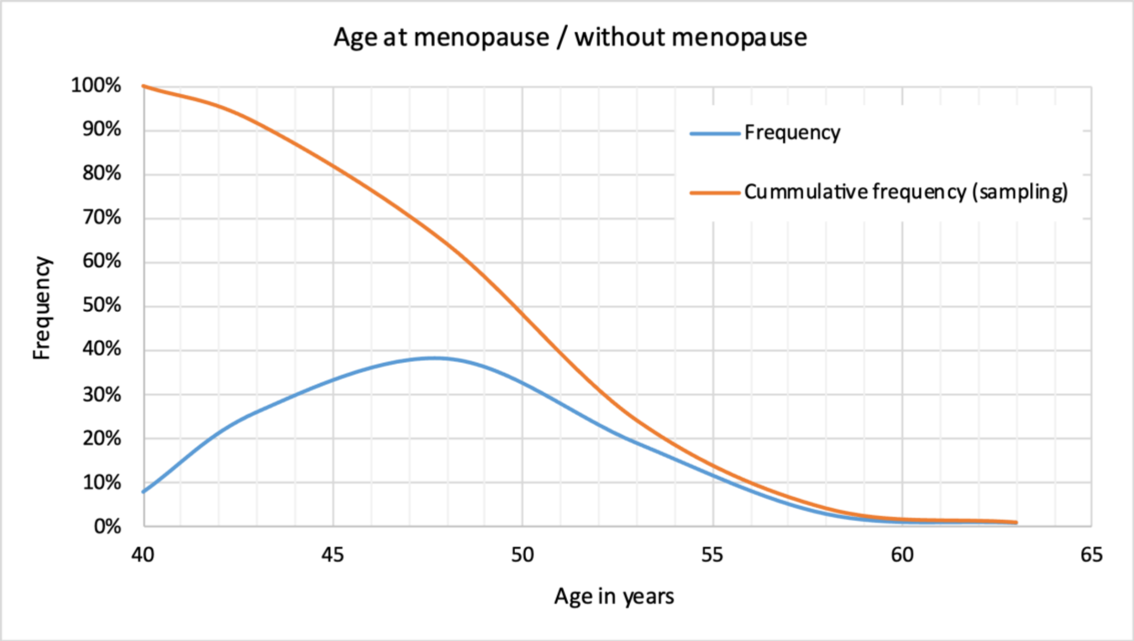

[3] Dr Judith Pearson, The National Migraine Centre. WH_May_WholeProof_p5.pdf (d2931px9t312xa.cloudfront.net)
[4] C. Stewart, 2023, Statistica.com Age at first experiencing menopause in the UK 2021 | Statista

Work productivity and activity impairment

The effects of eptinezumab on self-reported work productivity in adults with migraine participating in the DELIVER trial has been reported elsewhere.[5] However, the statistical relationship between absenteeism and presenteeism and migraine day frequency that was used in the model is presented in **Table S1**.

Table S1. Statistical description of work impairment on and off treatment

Variable	Coefficient (SE)	df	t-value	p-value
Absenteeism^{+}				
Intercept	6.04 (1.43)	897.3	4.21	<0.001
MMDs	0.89 (0.08)	1914	10.63	<0.001
Eptinezumab	–4.81 (1.41)	625.1	–3.41	<0.001
Presenteeism*				
Intercept	25.04 (2.51)	1290	9.97	<0.001
MMDs	4.78 (0.33)	3026.8	14.56	<0.001
MMDs^2	–0.09 (0.01)	2979	–6.96	<0.001
Eptinezumab	–10.78 (2.27)	656.6	–4.76	<0.001

Key: $^{+}$3,224 observations; *3,184 observations.
Abbreviations: ^2, squared; df, Degrees of freedom; MMDs, Monthly migraine days; SE, Standard error.

The relationship between absenteeism hours (y) and MMDs (x) was linear, given by the equation $y = b * x + c$, where b is the MMD coefficient and c is the intercept. For the eptinezumab strategy, the eptinezumab coefficient was added.

The relationship between presenteeism hours (y) and MMDs (x) was quadratic, given by the equation $y = -a * x^2 + b * x + c$, where a is the MMDs^2 coefficient, b is the MMD coefficient, and c is the intercept. For the eptinezumab strategy, the eptinezumab coefficient was added.

[5] Barbanti P, Awad SF, Regnier SA, Lee X, Goadsby PJ. Impact of eptinezumab on work productivity beyond reductions in monthly migraine days: Post hoc analysis of the DELIVER trial (P13-12.012). Neurology. 2023;100(17 Supplement 2):3020.

Input parameters

Input point estimates are detailed along with the selected parameter distribution and standard error used for probabilistic analysis (**Table S2**).

Table S2. List of input parameters

Area of input	Point estimate	Distribution for PSA	Standard error for PSA	Description	Source
Population	45.20	Excluded	N/A	Mean age of modelled population at commencement of treatment	DELIVER RCT TF3+ analysis (Data on file, Lundbeck A/S)
	11.84	Excluded	N/A	Standard deviation of modelled population at commencement of treatment	
	0.11	Excluded	N/A	Proportion of population who are male	
	0.00009	Excluded	N/A	Shape of Gompertz distribution for other cause mortality, females	ONS 2018-20 National life tables
	0.105	Excluded	N/A	Rate of Gompertz distribution for other cause mortality, females	
	0.00013	Excluded	N/A	Shape of Gompertz distribution for other cause mortality, males	
	0.105	Excluded	N/A	Rate of Gompertz distribution for other cause mortality, males	
	0.46	Excluded	N/A	Proportion of anti-CGRP population who experience CM	The Migraine Trust and Bionical Solutions (Report; The Migraine Patient Journey, UK)
Baseline MMD	9.76	Excluded	N/A	Alpha constant of beta distribution informing MMDs at baseline for EM	DELIVER RCT TF3+ analysis (Data on file, Lundbeck A/S)
	20.15	Excluded	N/A	Beta constant of beta distribution informing MMDs at baseline for EM	
	7.77	Excluded	N/A	Alpha constant of beta distribution informing MMDs at baseline for CM	
	4.44	Excluded	N/A	Beta constant of beta distribution informing MMDs at baseline for CM	
Effectiveness	0.096	Normal	0.020	Response rate for BSC ≥50% response rate over 12 weeks, EM	DELIVER RCT TF3+ analysis (Data on file, Lundbeck A/S)
	0.232	Normal	0.046	Response rate for BSC ≥30% response rate over 12 weeks, CM	
	0.400	Normal	0.080	Response rate for eptinezumab ≥50% response rate over 12 weeks, EM	
	0.643	Normal	0.129	Response rate for eptinezumab ≥30% response rate over 12 weeks, CM	
	–5.820	Normal	0.433	Change from baseline in MMDs vs eptinezumab for BSC ≥50% responder over 12 weeks, EM	
	–8.800	Normal	0.402	Change from baseline in MMDs vs eptinezumab for BSC ≥50% responder over 12 weeks, CM	

	–6.820	Normal	0.139	Change from baseline in MMDs vs eptinezumab for eptinezumab ≥50% responder over 12 weeks, EM	
	–9.930	Normal	0.168	Change from baseline in MMDs vs eptinezumab for eptinezumab ≥50% responder over 12 weeks, CM	
	–1.310	Normal	0.083	Change from baseline in MMDs vs eptinezumab for BSC ≥50% non-responder over 12 weeks, EM	
	–0.850	Normal	0.154	Change from baseline in MMDs vs eptinezumab for BSC ≥50% non-responder over 12 weeks, CM	
	–1.390	Normal	0.149	Change from baseline in MMDs vs eptinezumab for eptinezumab ≥50% non-responder over 12 weeks, EM	
	–1.710	Normal	0.365	Change from baseline in MMDs vs eptinezumab for eptinezumab ≥50% non-responder over 12 weeks, CM	
Natural history	49.500	Normal	9.900	Mean age of menopause onset in UK women 2022	Pokoradi 2011. RCGP survey of UK women
	5.000	Normal	1.000	Standard deviation in of mean age of menopause onset in UK women 2022	
	4.300	Normal	0.860	Mean duration of menopause symptoms, UK women 2022	Paramsothy 2017. US women
Waning	0.330	Normal	0.066	Years of MMD waning of active treatment effect before return to baseline level	Raffaelli 2020. Longitudinal cohort study, German
	1.000	Normal	0.200	Years of MMD waning of BSC effect before return to residual level	NICE appraisal precedence TA659
Discontinuations	0.003	Normal	0.001	Probability during initial assessment of experiencing a TEAE leading to eptinezumab withdrawal	DELIVER, All patients treated set (APTS)
	0.032	Normal	0.006	Exponent of distribution describing eptinezumab discontinuation due to TEAEs after initial assessment	Estimate based on extrapolation from the PREVAIL RCT
	0.300	Normal	0.060	Exponent of distribution describing eptinezumab discontinuation due to other causes	Calibrated
	1.250	Normal	0.250	Years between assessments for sustained response	Sacco 2022. EHF guidelines expert consensus statement 4
	3.000	Normal	0.600	Average MMDs for those in the Resolved health sate	Assumption
	4.000	Normal	0.800	MMD leeway below licensed MMD range before discontinuation	Assumption
HRQoL	0.225	Multi-normal	0.022	Intercept for MMD frequency disutility distribution when ON active treatment	DELIVER RCT TF3+ analysis (Data on file, Lundbeck A/S)
	0.013	Multi-normal	0.001	Slope constant for MMD frequency disutility distribution when ON active treatment	
	0.280	Multi-normal	0.028	Intercept for MMD frequency disutility distribution when OFF active treatment	

	0.013	Multi-normal	0.001	Slope constant for MMD frequency disutility distribution when OFF active treatment	
	–0.005	Beta	0.001	Disutility associated with each administration of eptinezumab applied every 12 weeks	NICE Eren TA682 (Vignette, scenario analysis)
Resources	0.132	Gamma	0.026	Hospital admissions in previous 6 months for MMD frequency 0–3	Report for Lundbeck A/S (v1.2). The prevalence and the Burden of Migraine in Europe (France, Germany, Italy, Spain, and United Kingdom) - A Population-Based Cross-Sectional Survey using the 2020 National Health and Wellness Survey (NHWS). Report on file.
	0.157	Gamma	0.031	Hospital admissions in previous 6 months for MMD frequency 4–7	
	0.188	Gamma	0.038	Hospital admissions in previous 6 months for MMD frequency 8–14	
	0.204	Gamma	0.041	Hospital admissions in previous 6 months for MMD frequency 15–30	
	0.258	Gamma	0.052	Emergency department visits in previous 6 months for MMD frequency 0–3	
	0.303	Gamma	0.061	Emergency department visits in previous 6 months for MMD frequency 4–7	
	0.299	Gamma	0.060	Emergency department visits in previous 6 months for MMD frequency 8–14	
	0.349	Gamma	0.070	Emergency department visits in previous 6 months for MHD frequency 15–30	
	0.747	Gamma	0.149	General practitioner visits in previous 6 months for MMD frequency 0–3	
	0.751	Gamma	0.150	General practitioner visits in previous 6 months for MMD frequency 4–7	
	0.769	Gamma	0.154	General practitioner visits in previous 6 months for MMD frequency 8–14	
	0.827	Gamma	0.165	General practitioner visits in previous 6 months for MMD frequency 15–30	
	0.083	Gamma	0.017	Neurologist visits in previous 6 months for MMD frequency 0–3	
	0.107	Gamma	0.021	Neurologist visits in previous 6 months for MMD frequency 4–7	
	0.158	Gamma	0.032	Neurologist visits in previous 6 months for MMD frequency 8–14	
	0.232	Gamma	0.046	Neurologist visits in previous 6 months for MMD frequency 15–30	
	0.056	Gamma	0.011	Psychiatrist visits in previous 6 months for MMD frequency 0–3	
	0.076	Gamma	0.015	Psychiatrist visits in previous 6 months for MMD frequency 4–7	
	0.079	Gamma	0.016	Psychiatrist visits in previous 6 months for MMD frequency 8–14	
	0.131	Gamma	0.026	Psychiatrist visits in previous 6 months for MMD frequency 15–30	
	0.100	Gamma	0.020	Primary care nurse visits in previous 6 months for MMD frequency 0–3	
	0.137	Gamma	0.027	Primary care nurse visits in previous 6 months for MMD frequency 4–7	
	0.116	Gamma	0.023	Primary care nurse visits in previous 6 months for MMD frequency 8–14	

	0.181	Gamma	0.036	Primary care nurse visits in previous 6 months for MMD frequency 15–30	
	1.000	Gamma	0.200	Manual adjuster of WPAI lookup table of days lost	
Unit costs	1850.000	Gamma	370.000	Monthly salary in the UK in 2020	Office for National Statistics, August 2020
	39.230	Gamma	7.846	Unit cost of a general practitioner visit	Personal Social Services Research Unit (PSSRU) 2020 Handbook
	196.630	Gamma	39.326	Unit cost of emergency department visit	NHS Reference costs 2019/20 (National cost collection data)
	567.990	Gamma	113.598	Unit cost of a hospital admission	
	40.000	Gamma	8.000	Unit cost of a nurse practitioner consultation	Personal Social Services Research Unit (PSSRU) 2020 Handbook
	187.170	Gamma	37.434	Unit cost of a neurologist consultation	NHS Reference costs 2019/20 (National cost collection data)
	187.170	Gamma	37.434	Unit cost of a psychiatrist consultation	
	7.280	Gamma	1.456	Unit cost of triptan (per MMD)	NICE TA631 Fremanezumab
	174.040	Gamma	34.808	Annual administration cost of eptinezumab 100 mg	NICE TA195 TNF inhibitors
	1835.712	Gamma	258.789	Annual WPAI earnings cost – eptinezumab, absenteeism, non-responders	DELIVER RCT
	507.236	Gamma	121.783	Annual WPAI earnings cost – eptinezumab, absenteeism, responders	
	9009.482	Gamma	426.240	Annual WPAI earnings cost – eptinezumab presenteeism, non-responders	
	4082.044	Gamma	487.131	Annual WPAI earnings cost – eptinezumab, presenteeism, responders	
	2777.722	Gamma	289.234	Annual WPAI earnings cost – BSC, absenteeism, non-responders	
	2198.024	Gamma	593.691	Annual WPAI earnings cost – BSC, absenteeism, responders	
	10168.879	Gamma	350.126	Annual WPAI earnings cost – BSC, presenteeism, non-responders	
	7898.393	Gamma	745.920	Annual WPAI earnings cost – BSC, presenteeism, responders	
	3091.726	Gamma	618.345	Annual WPAI earnings cost – BSC, absenteeism, refractory	
	12487.674	Gamma	2497.535	Annual WPAI earnings cost – BSC, presenteeism, refractory	
Drug price	1350.000	Excluded	N/A	Product unit cost of eptinezumab 100 mg	BNF online (Accessed June 2022)
